# Co‐Encapsulation of Probiotics and Prebiotics: Techniques and Applications in Food Fortification

**DOI:** 10.1002/fsn3.70426

**Published:** 2025-06-18

**Authors:** D. S. Shanuke, Nilmini Buddhika D. P. Ranasinghage, Ashinshana U. Illippangama, Jayani Kulathunga, Mithila D. Bandara

**Affiliations:** ^1^ Department of Food Technology, Faculty of Technology Rajarata University of Sri Lanka Mihintale Sri Lanka; ^2^ Department of Multidisciplinary Studies, Faculty of Urban and Aquatic Bioresources University of Sri Jayawardenepura Nugegoda Sri Lanka

**Keywords:** co‐encapsulation, dietary fibers, prebiotics, probiotics, wall material

## Abstract

There has been a growing interest in food products containing probiotics and prebiotics with synergetic effects. However, the primary challenge in fortifying food products with probiotics and prebiotics is maintaining their viability during processing and during passage through the digestive system. Co‐encapsulation has been identified as an effective approach to this issue. However, co‐encapsulation of probiotics and prebiotics and their applications warrant further exploration, focusing on the survival and functionality of prebiotics and probiotics. Therefore, this review aims to highlight various co‐encapsulation techniques and their impact on the survival and functionality of prebiotics and probiotics. Further, different types of prebiotics and probiotics that can be co‐encapsulated, types of polysaccharides and proteins that can be used as wall materials in co‐encapsulation, and the applications in food fortification in different food products.

## Graphical abstract

This review explores various co‐encapsulation techniques and their impact on the survival and functionality of probiotics and prebiotics in food systems. It discusses the types of probiotics and prebiotics suitable for co‐encapsulation, the polysaccharides and proteins commonly used as wall materials, and the potential applications of these systems in food fortification.

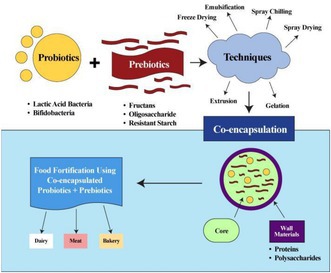

## 1 Introduction

In recent years, there has been a growing interest in functional food products with potential health benefits, particularly those incorporating bioactive compounds, prebiotics, and probiotic microorganisms (Rashidinejad 2024). According to the World Health Organization (WHO) and the Food and Agriculture Organization of the United Nations (FAO), probiotics are defined as living organisms that, when administered in an adequate amount, confer a health benefit on the host (FAO 2001). A daily consumption of 10^8^ to 10^9^ CFU/g (Colony Forming Units per gram) of probiotic viable cells is accepted by the scientific community as being the minimum amount required to confer the desired benefits (Haffner et al. 2016). *Lactobacillus* and *Bifidobacterium* strains are the most common probiotic microorganisms, and these beneficial bacteria have been associated with improved digestion, enhanced immune function, and reduced risk of certain diseases (Kent and Doherty 2014; Kerry et al. 2018). In contrast, prebiotics are non‐digestible food ingredients that selectively stimulate the growth and activity of beneficial bacteria already residing in the colon by providing a favorable environment for beneficial microorganisms that contribute to maintaining a healthy gut microbiota (Davani‐Davari et al. 2019; Klindt‐Toldam et al. 2016). Moreover, prebiotics exhibit a paramount of health‐promoting benefits, including their effects against acute gastroenteritis, constipation, diabetes, various types of cancer, and immune diseases (Ashwini et al. 2019; Yahfoufi et al. 2018).

In the food industry, probiotic cultures are mostly supplied in dried or frozen forms, as either spray dried or freeze dried powders (Anal and Singh 2007). This is due to the challenge faced in the incorporation of probiotics into the functional food product development while keeping the probiotics active during processing and during passage through the host's digestive system. As a solution to this, encapsulation of probiotics has been introduced to remain protected by the wall material from the harsh gastrointestinal environment until they reach the target destination (colon), where they break down and release the probiotic bacteria (Chawda et al. 2017).

Encapsulation refers to a sophisticated packaging technique that involves enclosing active elements, such as bioactive compounds or substances, within minute capsules or particles. Co‐encapsulation is considered an encapsulation of two or more symbiotic bioactive compounds into a single matrix (Chen et al. 2017). Co‐encapsulation of different food components plays a critical role in the protection of the nutrients against unfavorable processes and storage conditions such as elevated temperatures, high humidity, high oxygen levels, certain pH values, and light exposure (Assadpour and Jafari 2019).

The controlled release mechanism of co‐encapsules enables the sustained delivery of the encapsulated substances, finding applications in various fields, including pharmaceuticals, food science, and cosmetics (Corbo et al. 2013). In recent years, the food industry has recognized the potential of co‐encapsulation technology to develop numerous functional foods with improved health benefits. However, it is established that prebiotics can affect and enhance human gastrointestinal health, either alone or in combination with probiotic microorganisms in the form of symbiotics. Furthermore, functional food with both probiotics and prebiotics may affect the preserving potential and organoleptic properties of food products, and they should remain in a metabolically active state during processing and storage. Thus, co‐encapsulation is developed to enhance the viability of probiotics by combining proper prebiotics in the same core with synergetic effects (Anal and Singh 2007; Haffner et al. 2016).

The co‐encapsulation of probiotics and prebiotics can be achieved through various technologies, including both high‐temperature processes, such as spray drying, and low‐temperature processes, such as spray chilling, freeze drying, emulsification, extrusion, coacervation, gelation, and electro‐hydrodynamic atomisation. Moreover, different encapsulating materials are also used in the industry, such as alginate (Ding and Shah 2007), whey proteins (Doherty et al. 2015), pectin (Vaziri et al. 2018), and lipid carriers (Okuro et al. 2013). By incorporating co‐encapsulated prebiotics and probiotics, food products can be fortified with beneficial components, offering consumers an easy and convenient way to improve their gut health (Ozer et al. 2005).

Several reviews focusing on the encapsulation of probiotics or prebiotics have previously been published (Barajas‐Álvarez et al. 2023; Burgain et al. 2011; Frakolaki et al. 2020; Vivek et al. 2023). However, most of the previous reviews were focused on co‐encapsulation in general, and only a few comprehensive reviews were focused on the application of co‐encapsulation of probiotics with prebiotics (Rashidinejad et al. 2022). There is inconsistency in the literature, which may be related to the co‐encapsulation of probiotics with prebiotics using different techniques with their process parameters and specific conditions. Further, the co‐encapsulation of probiotics and prebiotic dietary fibers and their applications warrant further exploration focusing on the survival and functionality of prebiotics and probiotics. Therefore, this review article aims to provide a comprehensive overview of the co‐encapsulation of probiotics and prebiotics dietary fibers with different techniques, process parameters, conditions, and their applications in the food fortification of functional food products. We will further highlight various co‐encapsulation materials and techniques and their impact on the survival and functionality of prebiotics and probiotics.

## 2 Dietary Fibers and Their Importance in Gut Microbiota

The collection of densely populated microbial communities colonizing the gastrointestinal tract is termed the ‘gut microbiota’. The stomach, small intestine, and large intestine host essential resident microbial communities crucial for maintaining human health. The reports indicate that the human colon contains an estimated 10^10^–10^12^ living bacteria per gram, predominantly anaerobes residing in the large intestine (Collins and Reid 2016; Davani‐Davari et al. 2019).

The different species of microbes in the gut provide metabolic, immunologic, and protective functions that play a crucial role in human health (Holscher 2017). The gastrointestinal microbiota is influenced by several factors, including genetics, host physiology (age of the host, disease, stress, etc.), and environmental factors such as living conditions and use of medications (Goldsmith and Sartor 2014). Moreover, diet has been understood as a significant factor that influences the composition and metabolic activity of the gut microbiota. In fact, one way to influence the gut microbiota is to consume dietary fiber (Holscher 2017).

Dietary fibers are defined as the edible parts of plants or analogous carbohydrates that are resistant to digestion and absorption in the human small intestine with complete or partial fermentation in the large intestine (Tejada‐Ortigoza et al. 2015). Therefore, the dietary fibers are subjected to bacterial fermentation and thus impact the composition of bacterial communities as well as microbial metabolic activities, including the production of fermentative end products (Holscher 2017). Some of the dietary fibers can also be classified as prebiotics. Several factors affect how the gut microbiota uses dietary fiber, including the source of dietary fibers, type of molecules, bonds, chain length, particle size, and association with other compounds (Mcrorie and Fahey 2013).

The fermentation of dietary fibers by bacteria depends on their physicochemical properties, dosage, and the individual's bacterial community composition (Makki et al. 2018). Certain bacteria act as predominant dietary fibber degraders, producing acetate, propionate, and butyrate (Louis and Flint 2016) which, in turn, influence gene expression, energy metabolism, and act as signaling molecules regulating the immune system and inflammation (Koh et al. 2016; Louis and Flint 2016). Moreover, prebiotic dietary fibers can exert effects beyond the gastrointestinal tract. Short Chain Fatty Acids (SCFAs) produced during fermentation can permeate the blood circulation through enterocytes, influencing remote site organs (den Besten et al. 2013). Specific probiotic strains may also play a role in lowering the colonization of pathogens like *Staphylococcus aureus* and *Clostridium difficile* in healthy individuals, which supports the use of probiotics to prevent intestinal infections (Davani‐Davari et al. 2019).

## 3 Prebiotic Dietary Fibers

Prebiotics are defined as “non‐digestible food ingredients that beneficially affect the host by selectively stimulating the growth and/or activity of one or a limited number of bacteria in the colon, thus improving host health” (Davani‐Davari et al. 2019; Klindt‐Toldam et al. 2016). This definition was later refined to include other areas that may benefit from selective targeting of microorganisms, “a selectively fermented ingredient that allows specific changes, both in the composition and/or activity in the gastrointestinal microbiota that confers benefits”. Currently, the target genera are lactobacilli and bifidobacteria; however, prebiotic success has primarily been achieved with bifidobacteria. This may be due to the fact that more bifidobacteria usually reside in the human colon than lactobacilli, and they exhibit a preference for oligosaccharides (Brownawell et al. 2012). In order to characterize an ingredient as a prebiotic, it should have the ability to resist gastric acidity, hydrolysis by mammalian enzymes, and absorption in the upper GI tract, ferment by the intestinal microbiota, and selectively stimulate the growth and/or activity of intestinal bacteria potentially associated with health and well‐being. Moreover, all prebiotics can be classified as fibers, but not all fibers are prebiotics (Guarino et al. 2020). The most common types of prebiotics include fructo‐oligosaccharides (FOS), galacto‐oligosaccharides (GOS), isomalto‐oligosaccharides (IMO), xylo‐oligosaccharides (XOS), as well as polysaccharides found in plant cell walls.

### 3.1 Fructans

Fructans mainly consist of inulin and fructo‐oligosaccharides or oligofructose. Their structure is a linear chain of fructose units with a β‐(2→1) linkage. They usually have terminal glucose units with a β‐(2→1) linkage. Fructo‐oligosaccharides (FOS) have a DP of < 10, whereas inulin has a DP of up to 60. According to previous studies, fructans can selectively stimulate lactic acid bacteria. However, studies report that the chain length of fructans is one of the most important factors determining which bacteria can ferment them (Scott et al. 2014).

### 3.2 Galacto‐Oligosaccharide

Galacto‐oligosaccharides (GOS) are produced as a product of lactose extension. GOS are categorized into two subgroups, including the GOS with excess galactose at C3, C4, or C6 and the GOS produced from lactose via enzymatic transglycosylation. The end of this reaction results in a mixture of tri‐ to pentasaccharides with galactose in β‐(1→6), β‐(1→3), and β‐(1→4) linkages. These resulting GOS are also known as trans‐galacto‐oligosaccharides or TOS (Gibson et al. 2004; Macfarlane et al. 2008). Moreover, GOS can stimulate both Bifidobacteria and Lactobacilli. Especially in infants, Bifidobacteria have demonstrated a high affinity for GOS. In addition to that, GOS can also stimulate Enterobacteria, Bacteroidetes, and Firmicutes. However, this stimulation is comparatively lower than that observed in the Bifidobacteria (Louis et al. 2016).

### 3.3 Resistant Starch and Glucose‐Derived Oligosaccharides

The portion of starch that resists digestion as it passes through the gastrointestinal tract is known as resistant starch (RS), and it is classified as prebiotic due to its ability to promote health by producing a high level of butyrate (Munir et al. 2024; Zheng et al. 2020). The studies report the incorporation of different *Firmicutes* with RS. Furthermore, *Ruminococcus bromii* , *Bifidobacterium adolescentis* , *Eubacterium rectale*, and *Bacteroides thetaiotamicron* (Costabile et al. 2012). However, in the absence of *R. bromii* , RS degradation is impossible in mixed bacterial and fecal incubations (Costabile et al. 2012). IMO is considered a glucosyl saccharide containing one or more α‐(1,6)‐glycosidic linkages with or without α‐(1,4)‐glycosidic linkages. IMO is able to facilitate the proliferation of gut microflora in the colon, such as *Bifidobacterium* and *Lactobacillus*, by serving as the substrate to produce short‐chain fatty acids (Ranasinghage et al. 2023).

### 3.4 Other Oligosaccharides

There are oligosaccharides made of polysaccharides. These compounds are classified as pectic oligosaccharides (POS), and their structures are mainly dependent on their sources. The structures of POS are based on the extension of galacturonic acid (homogalacturonan) or rhamnose (rhamnogalacturonan I). In their structures, carboxyl groups may be substituted with methyl esterification or acetylated at C2 or C3. Different sugar types, including arabinose, galactose, xylose, and ferulic acid, can be linked to the side chains (Yoo et al. 2012). Additionally, polydextrose, which is like glucose‐derived oligosaccharides, is also considered a prebiotic. Polydextrose consists of branched glucans bound with glycosidic linkages. They are considered to have the possibility to stimulate Bifidobacteria (Costabile et al. 2012).

## 4 Probiotics

Probiotics are living organisms that, upon ingestion in certain numbers, exert health benefits beyond inherent basic nutrition (Kerry et al. 2018; Kim et al. 2017). Also, the term probiotics is defined as “live microorganisms that, when administered in adequate amounts, confer a health benefit on the host” (Kailasapathy 2009; Sandoval‐Mosqueda et al. 2019). The microorganisms such as *Bifidobacterium* spp., *Lactobacillus* spp., and *Enterococcus* spp., and some yeasts such as *Saccharomyces* spp., are mostly known as probiotics for their health‐promoting activities, besides their uses in food science (Doron and Snydman 2015). However, the most common probiotic types are bifidobacterial. Bifidobacteria have mostly been more successful than Lactobacilli due to their outnumbered residence in the human colon and affinity towards oligosaccharides (Brownawell et al. 2012).

### 4.1 Lactic Acid Bacteria

Lactic acid bacteria are usually gram‐positive, rod‐shaped, non‐spore‐forming, catalase‐negative organisms that are devoid of cytochromes and are of non‐aerobic habit but are aerotolerant, fastidious, acid‐tolerant, and strictly fermentative; lactic acid is the major end‐product of sugar fermentation. Some of the known Lactobacilli that are used as probiotics are *Lactobacillus acidophilus* , *Lactobacillus amylovorous*, *Lactobacillus casei* , *Lactobacillus crispatus* , *Lactobacillus delbrueckii* , *Lactobacillus gasseri* , *Lactobacillus johnsonoo*, *Lactobacillus paracasei* , *Lactobacillus plantarum* , *Lactobacillus reuteri* , *Lactobacillus rhamnosus* , *Lactobacillus plantarum* , *Lactobacillus acidophilus* , *Lactobacillus fermentum* , *Lactobacillus gastricus* , *Lactobacillus bulgaricus* , etc. (Anal and Singh 2007).

### 4.2 Bifidobacteria

Bifidobacteria are also gram‐positive and rod‐shaped bacteria. But they are considered strictly anaerobic bacteria. These bacteria can grow at pH levels in the range 4.5–8.5. Bifidobacteria actively ferment carbohydrates, producing mainly acetic acid and lactic acid in a molar ratio of 3:2 (v/v). They do not produce carbon dioxide, butyric acid, or propionic acid. The most recognized species of bifidobacteria that are used as probiotic organisms are *Bifidobacterium adolescentis* , *Bifidobacterium animalis* , *Bifidobacterium bifidum* , *Bifidobacterium breve* , *Bifidobacterium infantis* , *Bifidobacterium lactis*, and *Bifidobacterium longum* (Anal and Singh 2007). The mentioned Bifidobacteria have been mostly incorporated into functional food formulations. Other than these bacteria, *Bacillus cereus* var. toyoi, *Escherichia coli* strain nissle, *Propioniobacterium freudenreichii*, and some types of yeasts, for example, *Saccharomyces cerevisiae* and *Saccharomyces boulardii*, have also been identified as having probiotic effects (Anal and Singh 2007). But the use of yeasts like Saccharomyces as probiotics is still limited.

Consuming probiotics lowers the risk of intestinal infection and associated inflammation by preventing pathogenic invasion, potentially delaying the development of colorectal cancer (Fong et al. 2020). Furthermore, probiotics can enhance intestinal microflora, reduce lactose intolerance, enhance immunomodulatory activity, lower serum cholesterol levels, reduce mutagenicity, ease vaginitis symptoms, lower GIT diseases, and have anticancer properties, among other health benefits (Fong et al. 2020).

## 5 Applications of Prebiotic Dietary Fibers and Probiotics in the Food Industry

Currently, the applications and benefits of different probiotic strains in research and the food industry, along with investigating new methods for delivering probiotic bacteria in the gut, have gained more attention and interest in the food, nutraceutical, and pharmaceutical industries (Rashidinejad 2024). Probiotics are part of the most fermented dairy products, non‐dairy fermented foods, and probiotic‐fortified foods. They possess some important functional attributes not only for basic nutritional requirements but also for the positive responses to clinical treatments against several disorders and diseases such as diabetes, obesity, bowel syndrome, and even different types of cancer, where there is an advancing arena of research (Kerry et al. 2018; Mohajeri et al. 2018).

The majority of foods used as probiotic supplements in the market come from dairy products, including ice cream, cheese, yoghurt, and others. In the meantime, vegetarianism and health conditions like lactose intolerance, milk protein allergies, and blood cholesterol control are driving up demand for non‐dairy goods (Majzoobi et al. 2019). Probiotic bread products are therefore seen as a new trend and a way to broaden the options for probiotic supplement products (Majzoobi et al. 2019). However, studies on the enrichment of probiotics included in baked products were still limited because these products are baked at high temperatures, where the probiotic life is lost (Dong et al. 2020).

Prebiotic dietary fibers are mostly used in the dairy and bakery industries for nutritional benefits and as a sugar replacement and fat replacement as well (Longoria‐Garcia et al. 2018; Terpou et al. 2019). In the bakery industry, prebiotic dietary fibers are used in the products to have a soft dough, higher spread, better color, or higher oil retention. Inulin is one of the most common prebiotics added to bakery products to slow their digestion rate. In chocolates, the addition of prebiotics is associated with altering heat resistance and sugar replacement (Singla and Chakkaravarthi 2017).

Symbiotics are formed when probiotics are combined with prebiotics, which supply the body with beneficial bacteria and also act as a nutritional source for them. Yoghurt, kefir, cultured buttermilk, cultured cream, film, jolk, and koumiss are examples of such symbiotics that are available in the market (Singla and Chakkaravarthi 2017).

However, it is now understood that prebiotics can affect and enhance human gastrointestinal health, either alone or in combination with probiotic microorganisms in the form of symbiotics. Thus, the researchers have focused on increasing the range of probiotic and prebiotic viability in both carrier products and in the GIT. The main challenge facing the incorporation of probiotics and prebiotics into the functional food product development is keeping them active during processing and passage through the host's digestive system. In this context, co‐encapsulation is an effective approach to enhance the viability of probiotic cells and prebiotics during processing, storage, subsequent consumption, and gastrointestinal transit (Krasaekoopt et al. 2003).

## 6 Co‐Encapsulation

**FIGURE 1:**
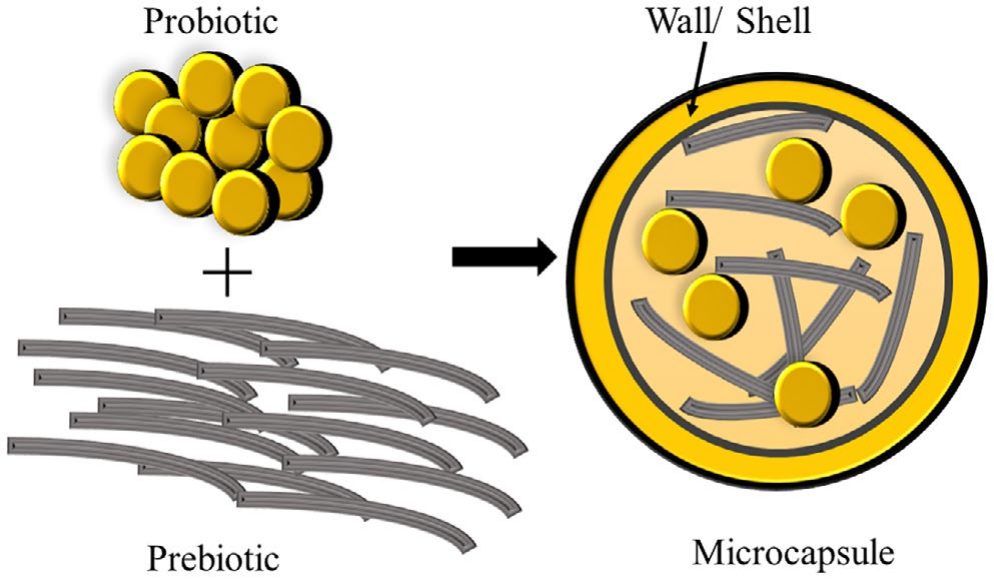
Co‐encapsulation of probiotic with prebiotic dietary fiber.

Co‐encapsulation refers to the encapsulation of two or more symbiotic bioactive compounds into a single matrix (Chen et al. 2017) providing protection and controlled release of encapsulated materials. This shields them from adverse reactions to lipid oxidation and nutritional deterioration during production, storage, and handling. It safeguards active ingredients from environmental factors and interactions with food components during gut passage, ensuring targeted delivery in functional and bioavailable form (Hashemi et al. 2014). Additionally, co‐encapsulation allows for targeted release at specific sites and can enhance organoleptic and flow properties (Hashemi et al. 2014). Co‐encapsulation techniques are used to encapsulate different combinations of essential oils, nutrients, and plant extracts that exhibit antimicrobial and antioxidant properties, vitamins, and different flavors (Fazilah et al. 2019; Phoem et al. 2019; Rashidinejad et al. 2022). Currently, co‐encapsulation technologies represent a prominent approach for probiotics and prebiotic encapsulation. The co‐encapsulation process involves enclosing prebiotics and probiotic bacteria inside a core, which is surrounded by a protective shell or wall. Figure 1 depicts the process of co‐encapsulation of probiotics with prebiotics. Similar to micro‐encapsulation, in co‐encapsulation, wall materials are dispersed to form the continuous phase, and the core consists of bioactive compounds. In the context of co‐encapsulation, probiotics or other active ingredients (prebiotics) are suspended or encapsulated within the continuous phase. There are several techniques used in co‐encapsulation, such as emulsification, spray drying, freeze drying, extrusion, coacervation, and electro spraying (Rashidinejad et al. 2022; Vázquez‐Maldonado et al. 2020; Zamora‐Vega et al. 2012).

### 6.1 Different Techniques of Co‐Encapsulation

There are several methods for co‐encapsulating probiotics with prebiotics. The selection of the co‐encapsulation method is crucial, as each method has both advantages and disadvantages. Therefore, it is important to be aware of the possible effects of the method on probiotics and prebiotics before making a choice. Widely applied techniques in probiotic and prebiotic co‐encapsulation include spray or freeze drying, spray chilling, extrusion, emulsification, and gelation.

#### 6.1.1 Spray Drying

**FIGURE 2:**
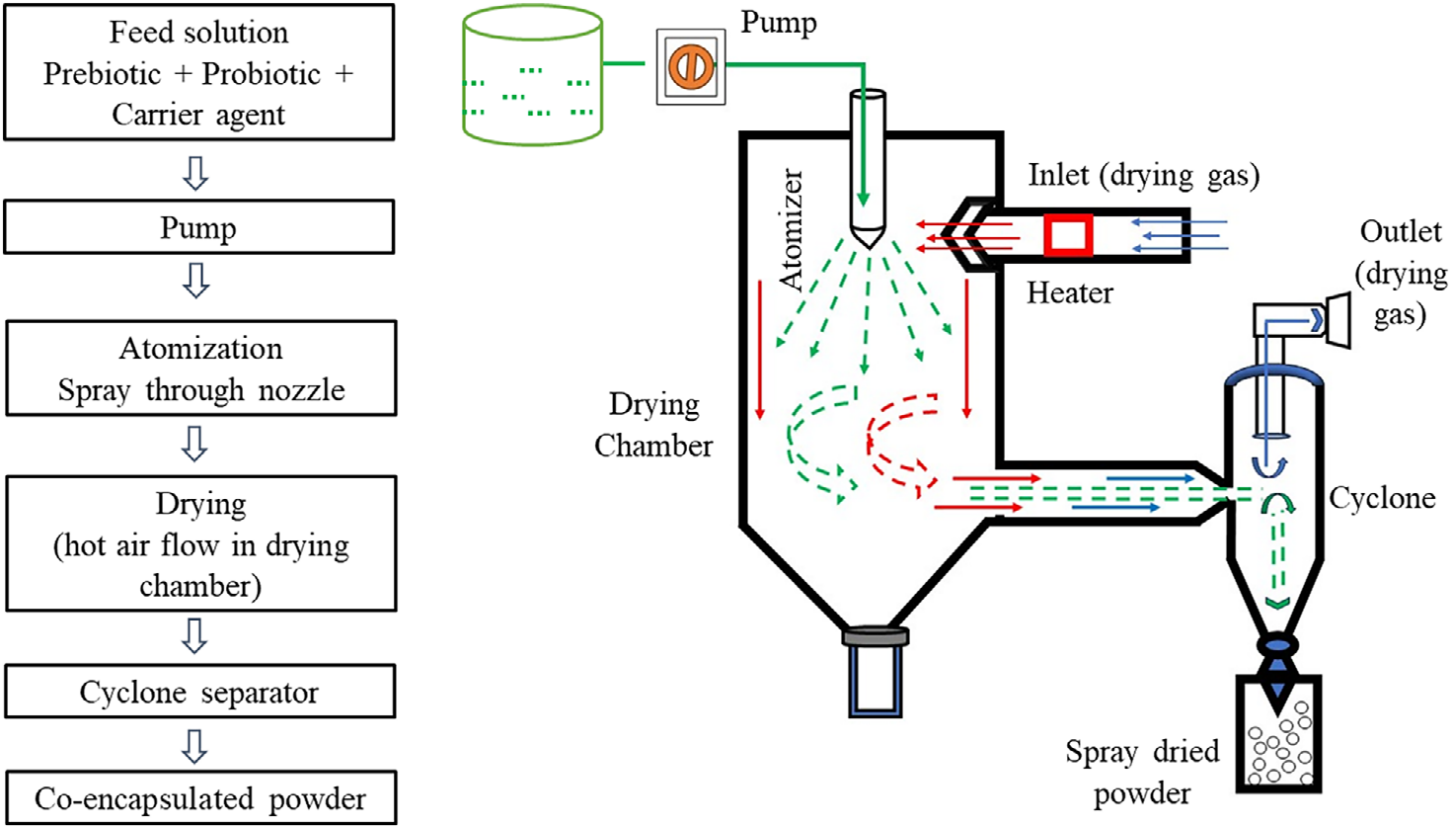
Schematic representation of spray drying process for co‐encapsulation of probiotics and prebiotics.

Spray drying, an extensively utilized microencapsulation technique, is particularly advantageous for co‐encapsulation applications. The spray drying process for probiotics involves several key components; these include a feed reservoir and pump, an atomizer, a heater and drying gas supplier, a drying chamber, a cyclone for particle separation, and an exhaust fan with a filter for drying gas removal (Assadpour and Jafari 2019). This method involves blending bioactive ingredients, such as prebiotics and probiotics, with a carrier solution. This feed solution is introduced into the atomizer through a peristaltic pump, where it is broken down into small droplets. These droplets are then directed into the drying chamber, where they come into contact with heated drying gas (typically air) supplied in parallel or counter‐current style (Figure 2). The hot gas quickly evaporates the moisture from the droplets, resulting in dried particles that fall to the bottom of the chamber (Assadpour and Jafari 2019). These particles contain the bioactive ingredients incorporated within the carrier material, designed to shield and effectively deliver them, offering potential health benefits and desirable functional properties in various applications. There are two types of spray dryers: standard spray dryers and nano spray dryers, producing particles within the micro‐scale and nano‐scale, respectively (Arpagaus et al. 2018; Assadpour and Jafari 2019).

Nano spray drying and conventional spray drying exhibit distinct advantages and limitations, particularly in particle size, scalability, encapsulation efficiency, and suitability for probiotic‐prebiotic co‐encapsulation. Both techniques offer benefits such as low cost, high equipment availability, process efficiency, reproducibility, and versatility. However, their differences significantly impact their industrial applications (Arpagaus et al. 2018).

Nano spray drying produces submicron particles (0.3–5 μm) with a narrow distribution (~1.4 μm), resulting in enhanced bioavailability, controlled release, and improved cellular penetration. These nanoparticles offer a higher surface‐to‐volume ratio, greater stability, and the potential for targeted delivery through surface modifications. Additionally, nano spray dryers utilize electrostatic collection, achieving high encapsulation efficiency (~93%) while maintaining the integrity of bioactive ingredients due to lower heat exposure and controlled laminar airflow. However, their small‐scale production capacity and inability to efficiently process high‐viscosity solutions limit their industrial feasibility (Assadpour and Jafari 2019).

Conversely, conventional spray drying generates larger microparticles (2–25 μm) with a broader size distribution (~1.8 μm). While this technique lacks efficiency in collecting particles smaller than 2 μm due to limitations in its cyclone separator, it remains the preferred method for large‐scale production. Its cost‐effectiveness and ability to handle higher viscosity solutions make it more suitable for industrial applications, with production capacities ranging from kilograms to tons of dried material (Piñón‐Balderrama et al. 2020). Furthermore, for probiotic‐prebiotic co‐encapsulation, conventional spray drying is the optimal choice since probiotic cells typically range from 1 to 5 μm, making them incompatible with nano spray drying. The process ensures probiotic viability, protects against environmental stress, and facilitates bulk production at a lower cost (Assadpour and Jafari 2019; Fathi et al. 2014).

While nano spray drying provides superior control over particle characteristics and bioavailability, its limited scalability and higher costs hinder its widespread industrial use. In contrast, conventional spray drying remains the preferred technique for large‐scale probiotic–prebiotic encapsulation, balancing efficiency, cost‐effectiveness, and industrial feasibility. The selection between these methods depends on specific formulation requirements, production scale, and economic considerations (Piñón‐Balderrama et al. 2020).

Widely used coating materials for encapsulating probiotics by spray drying encompass maltodextrin, modified starch, gum acacia, alginate, carboxymethyl cellulose, guar gum, soy protein, whey protein, and sodium caseinate. This process entails creating an emulsion by dispersing the core material within a polymeric solution, followed by homogenization. The resulting mixture is then atomized within a drying chamber to produce microcapsules of a matrix‐type structure. Encapsulation efficiency (EE) and particle size of co‐encapsulates in spray‐drying depend on various factors, including drying conditions and dispersion properties (Jafari et al. 2008). This continuous operation mandates meticulous control and adjustment of processing parameters, including flow rate, inlet and outlet temperatures, chamber temperature, and atomizer type to achieve the desired viability of the co‐encapsulated culture. Furthermore, emulsion viscosity and stability also affect the process. Spray drying involves atomizing liquid feed into fine droplets, typically ranging from 10 to 150 μm in size, which are subsequently introduced into a chamber containing hot, dry air at temperatures ranging from 150°C to 250°C. The ability of a probiotic strain to withstand osmotic, oxidative, and thermal stressors will determine its suitability for spray drying. Species such as *Lactobacillus*, *Lactococcus*, and *Bifidobacterium* display heightened sensitivity to spray drying conditions due to the application of elevated temperatures. The reduction in water activity during spray drying increases bacterial resistance to osmotic stress, with viability maintained by the buildup of suitable solutes such as carbohydrates, amino acids, or quaternary amines (Wood 2011). In comparison to freeze drying, spray drying consumes less energy and is an economically viable encapsulation method. Avila‐Reyes et al. (2014) used native rice starch and inulin as prebiotics to encapsulate *Lactobacillus rhamnosus* by spray drying at drying temperatures of 135°C, 145°C, and 155°C. The lowest reduction of probiotics was recorded by native rice starch added microcapsules spray dried at 135°C. *Lactobacillus plantarum* has been successfully co‐encapsulated with red beet stem extract using spray drying (de Deus et al. 2023). The highest encapsulation efficiency after spray drying was achieved by the treatment that contained 50% beet stem extract, and the viability of the bioactive compound and *Lactobacillus plantarum* was better preserved for 120 days (Yonekura et al. 2013). Co‐encapsulated Lactobacillus acidophilus NCIMB 701748 by spray drying using a carrier solution prepared from protein/carbohydrate and soluble fiber. The low moisture content and fine particles can be achieved by co‐encapsulation from spray drying (Fathi et al. 2014). Resveratrol and *Bacillus clausii* were co‐encapsulated via spray drying at 210°C inlet and 70°C outlet temperatures and 1.5 bar pressure. In this study, bacterial survivability in prebiotic lactose and inulin carriers was 8.62 and 8.52 log CFU/g, respectively (Vázquez‐Maldonado et al. 2020). Although spray drying is widely employed in co‐encapsulation, it has several limitations. One major drawback is the significant reduction in the viability of probiotic organisms due to exposure to high temperatures. Additionally, the potential degradation of heat‐sensitive bioactive compounds has not been fully addressed. The exclusive use of water‐soluble wall materials further restricts the incorporation of non‐water‐soluble materials, limiting their application. Moreover, the rapid drying process can lead to the formation of microcapsules with irregular, highly porous surfaces, which may adversely impact their morphology. This, in turn, results in increased core material release and reduced protection efficiency. Spray drying requires complex equipment, considerable installation space for industrial‐level applications, and high capital and maintenance costs associated with significant economic constraints, limiting its feasibility for certain applications (Barajas‐Álvarez et al. 2023; Rajam and Subramanian 2022; Rashidinejad et al. 2022).

#### 6.1.2 Spray Chilling

**FIGURE 3:**
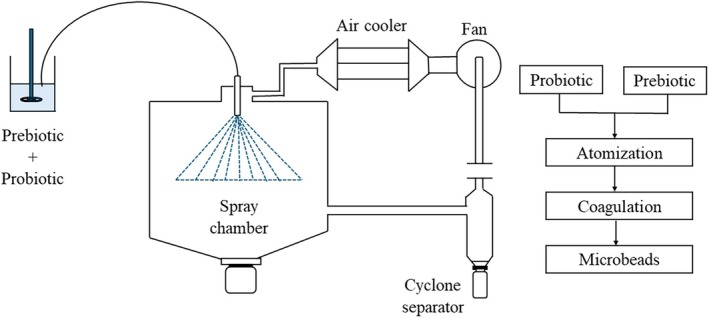
Schematic representation of spray chilling process for co‐encapsulation of probiotics and prebiotics.

Spray chilling, also referred to as spray cooling, exhibits a similarity to the process of spray drying. However, a key difference lies in the methodology of spray chilling, where atomization of the dispersion matrix occurs within a cold air or liquid nitrogen environment maintained below the melting point of the carrier and bioactive ingredients, as shown in Figure 3. In this method, lipids are widely used as the encapsulating material, and the core substance is first dispersed in a lipid matrix before being atomized in a chamber infused with cold air (Liu et al. 2017). During gastrointestinal transit, the release of probiotics happens when intestinal lipases digest the lipid wall after the carrier lipids melt when they reach their melting point. Spray chilling is ideal for controlled probiotic release, offering cost‐effectiveness with high yields in continuous or batch production. This technique is environmentally friendly due to mild processing conditions, low energy, and no heat requirement, thus retaining probiotic viability (Frakolaki et al. 2020; Singh et al. 2018). When compared to spray drying, spray chilling is a cost‐effective and scalable method, but the encapsulation efficiency is lower. Additionally, there are other disadvantages, such as active ingredient expulsion, instability, crystallization, poor protection of the core, and compromised release of active agents (Okuro et al. 2013; Wang and Zhong 2024). In a study, co‐encapsulation of probiotics, including *S. boulardii*, *L. acidophilus* , and *B. bifidum* , was conducted using spray drying and spray chilling methods. Encapsulation of probiotics by spray chilling was conducted using an emulsion prepared with hydrogenated palm oil as the carrier solution. Spray chilling was performed at room temperature while the temperature of the feeding solution was maintained at 45°C, the nozzle temperature was fixed at 38°C, and the air pressure was at 0.3 bar (Arslan‐tontul et al. 2019). In this study, spray drying was employed using a 9:1 ratio of gum arabic and β‐cyclodextrin as wall materials and performed using spray drying at an inlet temperature of 120°C and an outlet temperature of 50°C. Furthermore, double‐layered microcapsules were formed in this study by combining spray chilling and spray drying microencapsulation techniques to increase the survivability of probiotic microorganisms during the fortified cake baking process. Results showed that the double‐layered *S. boulardii* microcapsule (outer layer was spray chilled and inner layer was spray dried) showed the highest survivability in baked fortified cakes. *S. boulardii* was microencapsulated with different wall materials (gelatin, whey protein concentrate, modified starch, maltodextrin, pea protein isolate, and gum arabic) by spray drying (Arslan et al. 2015). However, there is a potential to combine spray chilling with other microencapsulating techniques to improve the survivability of different probiotics with different novel wall materials.

#### 6.1.3 Freeze Drying

**FIGURE 4:**
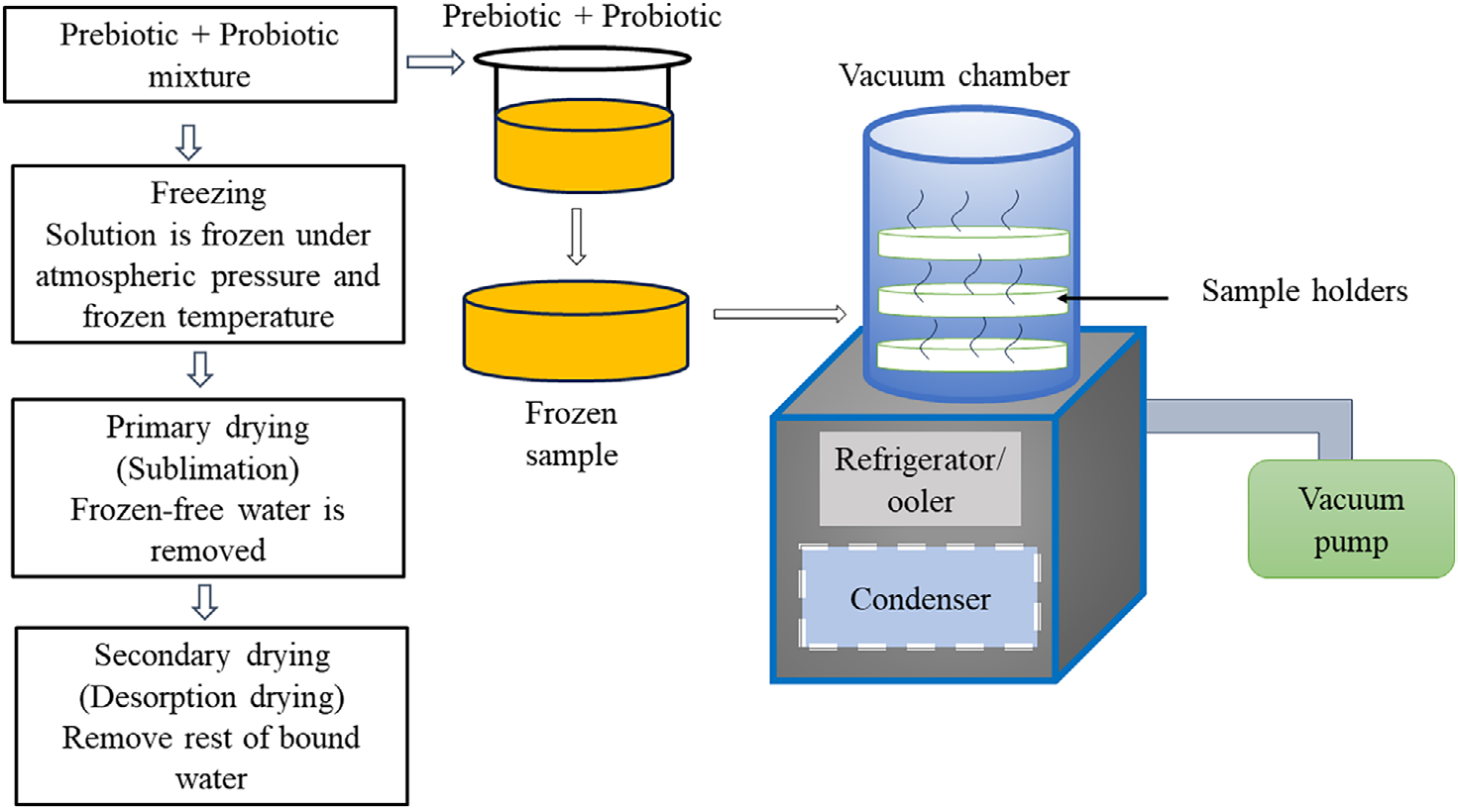
Schematic representation of freeze drying process for co‐encapsulation of probiotics and prebiotics.

Freeze drying technology holds the potential to make significant contributions across various fields, ranging from biopharmaceuticals to food science and health‐related disciplines. This technology relies on the process of sublimation, which entails converting frozen solutions or dispersions containing probiotics, prebiotics, other bioactive compounds, and carrier materials into vapor under high‐vacuum conditions (Figure 4) (Liu et al. 2017). Freeze drying can be implemented in two primary forms: direct freeze drying and indirect freeze drying. In the direct freeze drying method, probiotic bacteria are mixed with a cryoprotectant solution and then subjected to freeze drying. Conversely, in indirect freeze drying, probiotic bacteria are first encapsulated within a polymer matrix to create probiotic beads. These beads are subsequently freeze‐dried to eliminate water content (Liu et al. 2017). The process of freeze drying provides a wide range of practical uses, especially in better maintaining the vitality and effectiveness of probiotic microorganisms and prebiotic components. This is due to its lower operating temperature when compared to spray drying (Sharifi et al. 2020). Lengthy drying times (24–36 h), the need for complex equipment with limited process flexibility, high capital and maintenance costs, and reduced thermal efficiency significantly constrain the industrial‐scale application of freeze drying in co‐encapsulation processes (Rajam and Subramanian 2022).

Freeze‐dried co‐encapsulated *Lactobacillus casei* ssp. *paracasei* and black currant extract achieved high encapsulation efficiencies of 87.38% and 95.46%, respectively. The process utilized inulin, whey protein isolate, and chitosan as effective coating materials, and freeze drying conditions were maintained at 42°C under a pressure of 10 Pa for 48 h (Enache et al. 2020). However, it is possible to damage the probiotic cell wall due to the formation of extracellular ice crystals during freeze drying. The use of an appropriate cryoprotectant can protect probiotics bacteria's cell wall since cryoprotectants can stabilize the cell wall by interacting with the bound water of cells (Liu et al. 2017).

#### 6.1.4 Extrusion

**FIGURE 5:**
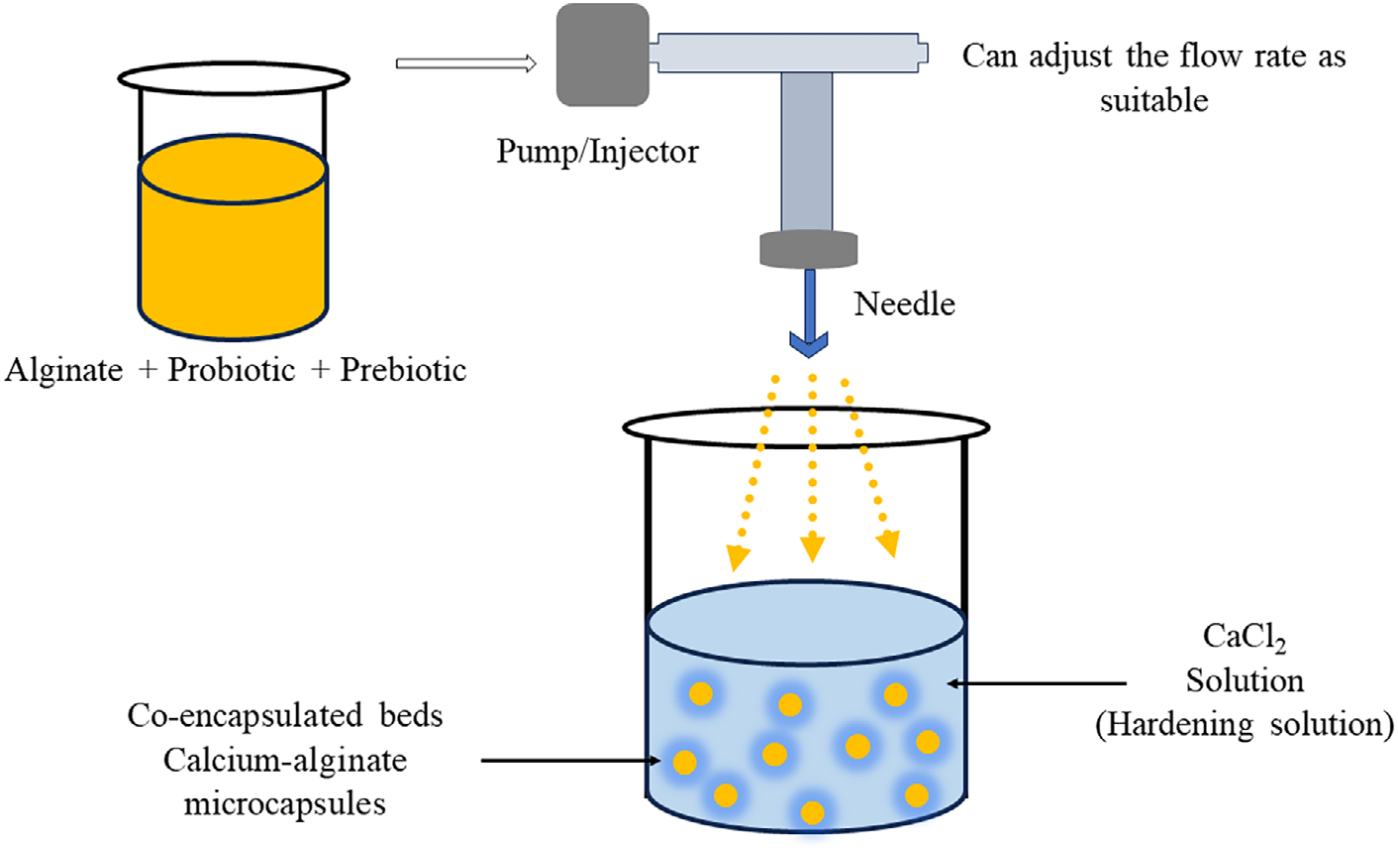
Schematic representation of the extrusion process for co‐encapsulation of probiotics and prebiotics.

Extrusion is a physical technique applied in microencapsulation and co‐encapsulation. High viability of probiotics could be achieved through extrusion since mild operational conditions are applied in this process. This process has three steps: creating a solution containing probiotics and prebiotics within a hydrocolloid. Subsequently, this solution is carefully introduced into a solidifying mixture through a syringe nozzle, and finally, the resulting beads are then subjected to a drying process as outlined in Figure 5. Alginate is widely used in extrusion, with calcium chloride commonly employed as the hardening solution. In the co‐encapsulation of probiotic bacteria in alginate is added to the hardening solution using a syringe with a needle. The size and characteristics of the microcapsules produced through the extrusion technique are impacted by several factors, such as the concentration of alginate, the diameter of the needle, the pressure applied to the syringe, the concentration of CaCl_2_, and the rate at which the solution containing alginate is agitated during the process of capsule formation (Phoem et al. 2019; Valero‐Cases and Frutos 2015). This approach avoids the use of harmful solvents and can be conducted in both aerobic and anaerobic settings. However, a notable drawback of this technique is its limited suitability for large‐scale manufacturing due to the gradual formation of the microbeads and large particle size. Co‐encapsulation of probiotic *Lactobacillus casei* TISTR 1463 with prebiotics such as IMO, maltitol, and tapioca‐based resistant starch in an alginate medium by extrusion was investigated (Apiwattanasiri et al. 2022). In this study, the suspension, with or without silk sericin, probiotics, and prebiotics, was extruded into a CaCl_2_ solution using an air pump equipped with an 18 G needle (1.2 × 40 mm). After the extrusion process, the formed microcapsules were freeze‐dried at 40°C under a pressure of 0.12 mbar for 12 h. According to the results, alginate‐silk sericin‐maltitol with silk sericin coating provided the best protection for probiotics, exhibiting the lowest inactivation rate and higher survival rates when exposed to high temperatures and digestive conditions. In another study, co‐encapsulation of *L. acidophilus* with apple skin polyphenols via co‐extrusion technology in milk showed minimal reduction in bacteria cells (0.13 log) after 50 days, with enhanced viability (> 10^6^ CFU/mL) (Shinde et al. 2013). The extrusion technique for co‐encapsulation of probiotics and prebiotics presents several challenges at an industrial scale. Scaling up the process is difficult due to the need for precise control over temperature, shear forces, and material consistency. High temperatures and mechanical stress during extrusion can harm the viability of probiotics, reducing their effectiveness and stability. Additionally, achieving a high core‐to‐wall material ratio is essential for cost‐effectiveness and product functionality. However, this ratio is hard to maintain during extrusion, leading to higher material costs. Furthermore, variations in the extrusion process can impact the sensory qualities of the final product, such as texture, flavor, and appearance, which may affect consumer acceptance. These factors make extrusion a less favorable option for large‐scale co‐encapsulation of probiotics and prebiotics. This can be improved by modifying the process with methods such as electrostatic air flow extrusion, rotating disk spraying, or using multiple nozzles simultaneously. These modifications help enhance particle size control and increase production speed (Agriopoulou et al. 2023; Jacobsen et al. 2020).

#### 6.1.5 Emulsification

**FIGURE 6:**
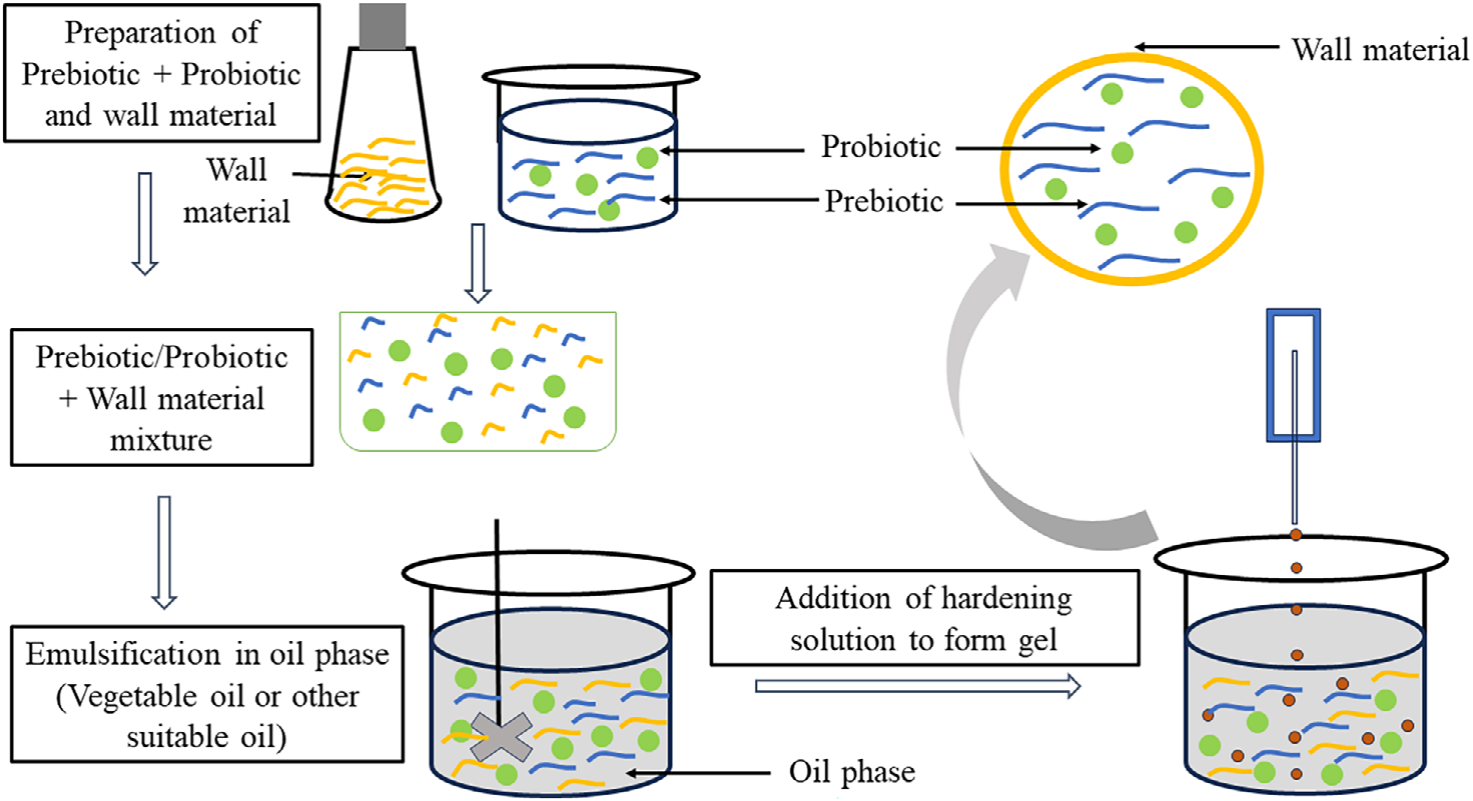
Schematic representation of the emulsification process for co‐encapsulation of probiotics and prebiotics.

Emulsification is a chemical technique used to encapsulate probiotic cells and use hydrocolloids as encapsulating materials (Figure 6). Generally, emulsions contain at least one immiscible phase dispersed into another continuous phase with a surfactant or emulsifier (Rezaei et al. 2019). There are three methods of emulsification for the co‐encapsulation of probiotic cells: emulsification and ionic gelation, emulsification and enzymatic gelation, and emulsification and interfacial polymerization (Burgain et al. 2011; Gaudreau et al. 2016). Co‐encapsulated *Saccharomyces boulardii* CDBB‐L‐1483 ATCC‐MYC‐797 with inulin by emulsification of aqueous alginate solution with canola oil. In this study, gelation was conducted by adding glacial acetic acid. As a result, alginate gels exhibited superior hardness compared to gels incorporating other hydrocolloids, signifying stronger gel formation. Blending alginates with mucilage and inulin enhanced yeast viability, with encapsulated yeast showing higher counts and viability than free yeast after 30 days at 48°C. Furthermore, over a storage period of 35 days, encapsulated yeast maintained 76.1% viability, surpassing the 63.3% viability of free yeast. Emulsification is applied in industrial‐scale encapsulation processes as a preparatory step for other techniques like ionic gelation, spray drying, etc. (Agriopoulou et al. 2023).

Surfactants play a fundamental role in the emulsification process by reducing interfacial tension between immiscible phases (typically oil and water) and stabilizing the resulting emulsion droplets. Their amphiphilic nature enables them to position themselves at the oil–water interface, decreasing the energy required to maintain dispersed droplets, preventing coalescence, and promoting the formation of fine, uniform emulsion droplets.

In probiotic encapsulation, stabilization of core materials using suitable surfactants is crucial since it creates a protective microenvironment that shields probiotic cells from external conditions such as heat, oxygen exposure, and pH variations, thereby enhancing cell viability during processing and storage (Camelo‐Silva et al. 2022). The emulsification technique stabilizes probiotics by embedding the live cells within these stabilized droplets, protecting them from mechanical and chemical degradation. This technique is more effective when combined with hydrocolloid‐based encapsulating agents such as alginate, chitosan, or inulin‐like materials that provide additional mechanical strength. The use of suitable surfactants helps the emulsion maintain strong structural integrity throughout subsequent processes such as ionic gelation or spray drying. Furthermore, surfactants can regulate the release of probiotics by controlling droplet size and the thickness of the encapsulating layer. A well‐stabilized emulsion ensures that probiotics, along with prebiotics in the core, remain viable during the production process and survive gastrointestinal conditions, allowing for targeted delivery to the intestines. Optimizing the type and concentration of surfactants is crucial to achieve high encapsulation efficiency, long‐term stability, and enhanced probiotic functionality in complex food and pharmaceutical formulations (Albadran et al. 2018; Camelo‐Silva et al. 2022).

#### 6.1.6 Coacervation

**FIGURE 7:**
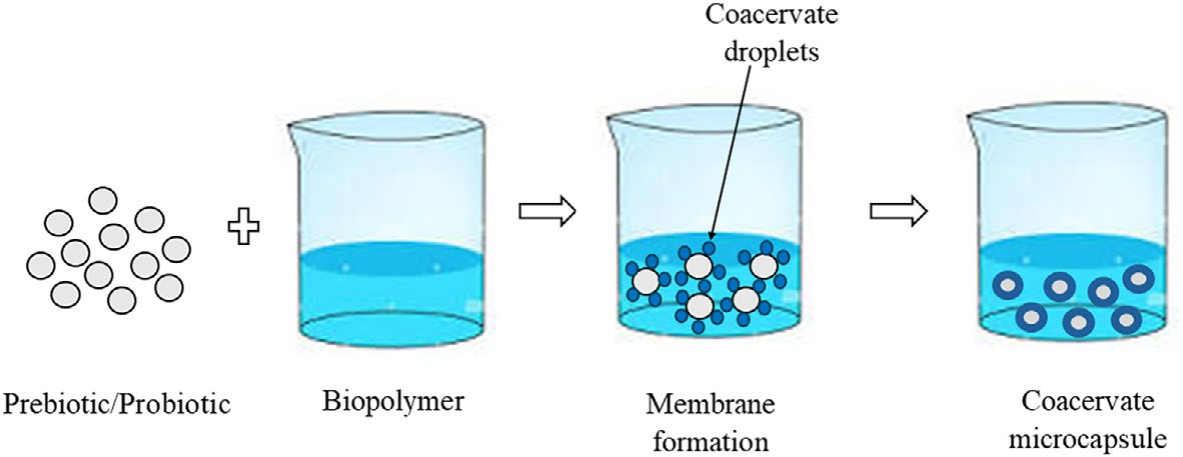
Schematic representation of the coacervation process for co‐encapsulation of probiotics and prebiotics.

Coacervation is a physical method based on electrostatic interactions between biopolymers with opposite charges that can be classified into two types: simple coacervation and complex coacervation (Ghasemi et al. 2018). The mechanism of simple coacervation involves the electrostatic interaction between a bioactive compound (either prebiotic or probiotic) that carries a charge and a biopolymer that carries an opposite charge, serving as the carrier for the compound (Figure 7). In contrast, complex coacervation is a phenomenon that involves the electrostatic interaction between two biopolymers possessing opposite charges. In this process, the carrier acts as a medium for encapsulating bioactive ingredients, such as prebiotics and probiotics. This process occurs through electrostatic interactions between molecules carrying opposite charges, resulting in the formation of compact and viscous coacervate phases. A combination of protein and polysaccharides with opposite charges is commonly used for the formation of complex coacervation (Ghasemi et al. 2018). Proteins such as whey protein, gelatin, albumin, and different plant proteins, along with polysaccharides like alginate, pectin, chitosan, and various gums like xanthan gum and carboxymethyl cellulose, have been utilized in the process (Eratte et al. 2015). During complex coacervation, the carboxyl groups present in polysaccharides interact with the amino groups of proteins, leading to the formation of a complex that includes an amide bond (Santos et al. 2015).

The final step is the hardening and filtering of microcapsules. The carrier material's surface charge affects the capsule's functional properties, which are influenced by various factors such as pH, temperature, and polymer concentration. After the formation of the complex coacervates, a drying method may be used to produce powder capsules. In comparison to other drying methods, spray drying and freeze drying are more suitable (Timilsena et al. 2018). *Lacticaseibacillus paracasei* BGP‐1 and *Bifidobacterium animalis* subsp. lactis BLC‐1 was encapsulated with guarana extracts by coacervation. In this study, guarana extract was used as a prebiotic, which increased the viability of probiotics. Coacervation of probiotics and guarana extract was performed by adjusting the pH of homogenized gelatin‐gum arabic suspension containing guarana extract and probiotics to 3.8 using citric acid. Prepared coacervates were freeze dried for 48 h, and co‐encapsulated probiotics and guarana extracts were able to improve the survival of cells after exposure to simulated intestinal fluid around 7 log CFU/mL (Silva Cristina et al. 2017). The main limitations of this process include the sensitivity of microcapsules to changes in pH or ionic strength, challenges in scaling up the method, and potential harm to probiotic cells during stages such as crosslinking and drying (Timilsena et al. 2018).

#### 6.1.7 Gelation

**FIGURE 8:**
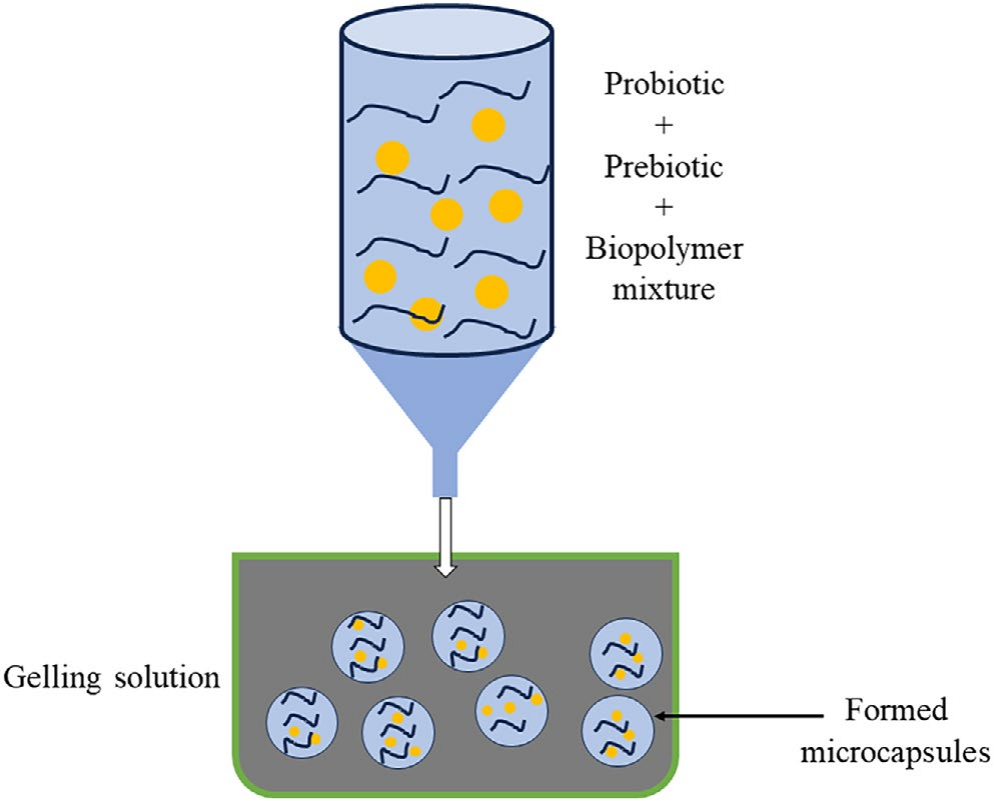
Schematic representation of gelation process for co‐encapsulation of probiotics and prebiotics.

Gelation technique can be used to encapsulate probiotics and prebiotics within different polymers, such as alginate, carrageenan, and sodium carboxymethyl cellulose (Figure 8). As an example, in the process of external gelation for alginate, the bioactive components are combined with an alginate solution to create a water–oil emulsion in oil containing an emulsifier. Alginate beads are produced by adding CaCl_2_ as which results in breaking the emulsion. In internal gelation, first, a water/oil emulsion is made by mixing alginate with CaCO_3_; then, organic acid is added to liberate calcium ions to produce an alginate gel (Serrano‐casas et al. 2017). Additionally, organogels or hydrogels produced from different polysaccharides are also used in encapsulation (Debele et al. 2016). *Lactobacillus acidophilus* was co‐encapsulated with FOS in alginate‐gelatin and alginate‐gelatin‐FOS microbeads through extrusion by external gelation with the aid of a double fluid atomizer nozzle with 0.7 mm diameter (Silva Cristina et al. 2017). Co‐encapsulation enhanced cell protection against SGI conditions by 20%. The inclusion of FOS in the matrix fostered the development of a more interconnected network, further enhancing cell protection and ensuring controlled delivery. Notably, the co‐encapsulation process had no adverse impact on cell viability. The primary limitation of microbeads is their poor stability in acidic environments, as their porous structure makes them vulnerable to diffusion from the surrounding solution. Additionally, the encapsulation process is time‐consuming and difficult to scale up for larger production (Yao et al. 2018).

#### 6.1.8 Electro‐Hydrodynamic Processes

**FIGURE 9:**
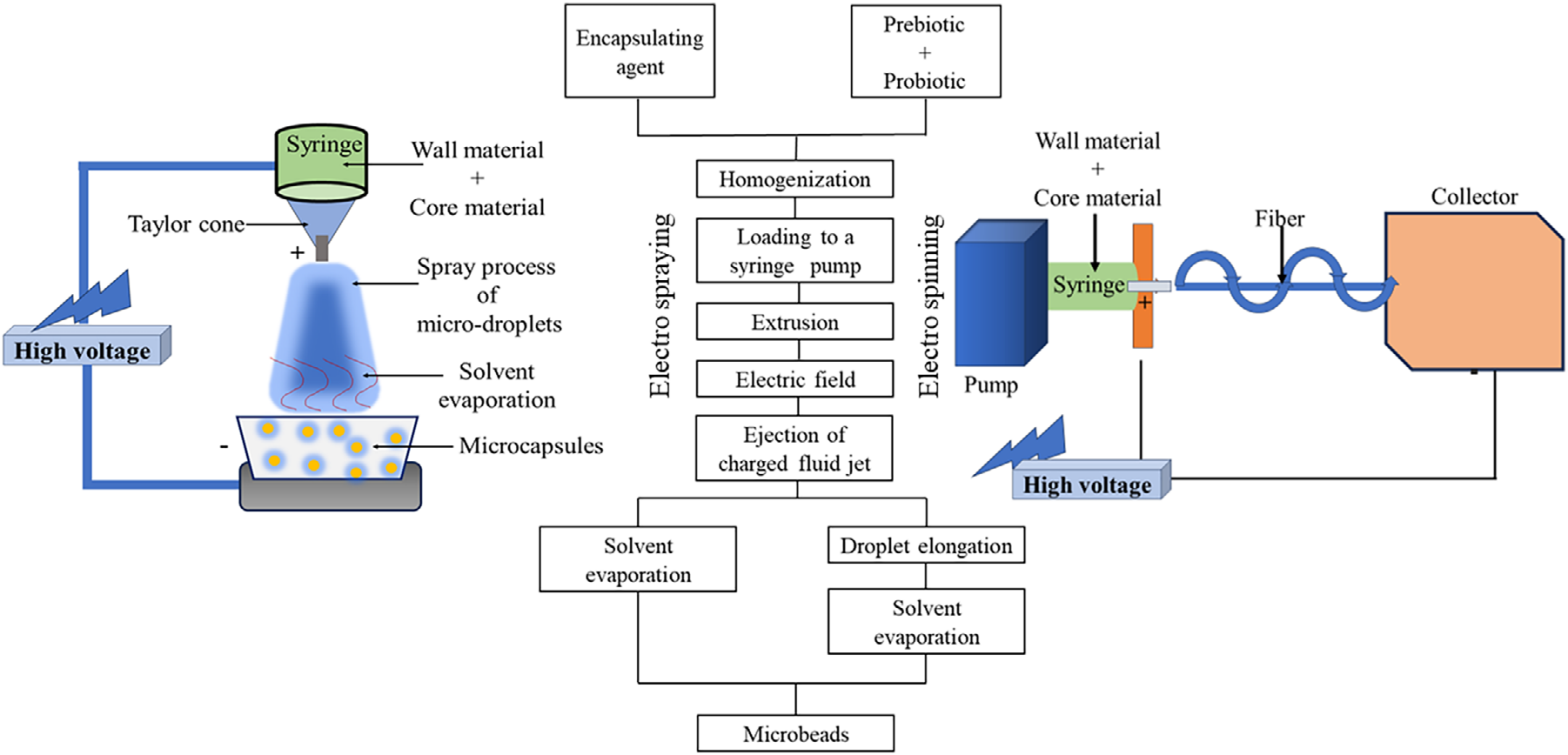
Schematic representation of electrospraying and electrospinning processes for co‐encapsulation of probiotics and prebiotics.

This method involves applying an external electric field between two electrodes to a biopolymer solution containing probiotic bacteria and prebiotics (Figure 9). Through the use of a syringe, the probiotic‐laden biopolymer solution is continuously pumped at a consistent rate, forming a droplet at the needle's tip. Voltage supplied to the equipment typically falls within the range of 5 to 30 kV. Upon application of the electric field, electrostatic repulsion of charges within the solution overcomes surface tension, resulting in the ejection of a jet. This jet moves towards the grounded cathode. Notably, this technology operates under mild conditions, devoid of harsh elements such as high temperatures, making it ideal for encapsulating sensitive, biocompatible, and biodegradable compounds, including probiotics (Vivek et al. 2023). The electrospray technique is employed in co‐encapsulation to combine two different materials, generally polymers or bioactive compounds, into micro‐ or nano‐droplets. By applying electrostatic forces to a polymer solution containing the materials of interest, the solution is atomized into ultrafine droplets. This process is called' electrospraying' nd another technique known as electrospinning is used in electro‐hydrodynamic processes to produce ultrafine fibers by spinning polymer solution. Resultant droplets can then be solidified to form particles or capsules that encapsulate both substances simultaneously, enabling controlled and efficient co‐encapsulation for various applications (Soleimanifar et al. 2020; Rezaei et al. 2015). Even though these electrohydrodynamic techniques require high capital costs, expensive installation, and advanced technology requirements, both electrospraying and electrospinning are emerging techniques used in industrial food processors to encapsulate probiotics, creating nano‐ and micro‐scale fibers. For instance, Xu et al. (2022) observed an 89.26% survival rate for *L. rhamnosus* 1.0320 after electrospinning and an 84.63% survival rate after 21 days of storage. When polyvinyl alcohol and pectin were used as the wall material in simulated gastric and intestinal conditions, survival rates were 90.07% and 91.96%, respectively. Xu et al. (2022) and Ma et al. (2023) improved the stability of *L. plantarum* KLDS 1.0328 using polyvinyl alcohol.

### 6.2 Different Wall Materials Used in Co‐Encapsulation

There are different wall materials, such as organic, inorganic, and combined wall materials made of an organic–inorganic combination. The wall could be formed using a single polymer or a combination of one or two polymers. The main functions of the wall are giving mechanical strength to microparticles as well as maintaining their shape (Alva et al. 2017). Thicker walls have better mechanical strength. The properties of the wall depend on the type of polymer chosen as wall material and also on conditions during the synthesis process. There are some facts to consider when choosing a wall material in co‐encapsulation. The wall should be a material with good chemical stability that does not react with the core. It is desirable to have a wall material with high thermal conductivity (Alva et al. 2017). The main food‐grade wall materials used for encapsulation and co‐encapsulation are polysaccharides, proteins, polymers, and lipids (Liu et al. 2022). Among them, various types of polysaccharides and proteins are widely used in the co‐encapsulation of probiotics and prebiotics. The co‐encapsulation process is usually performed in three stages. The first step is incorporating the bioactive component in a matrix (core). Then disperse core materials with the continuous phase, and finally, stabilization and drying (Rashidinejad et al. 2022). Suitable wall material should be carefully determined considering the prebiotic, probiotic, and method of encapsulation.

#### 6.2.1 Polysaccharides

##### 6.2.1.1 Alginate

Alginate is a naturally derived linear heteropolysaccharide in various algae, which consists of β‐d‐manuronic acid and α‐l‐guluronic acid. Therefore, alginate is an unbranched polymer. The ratio between β‐d‐manuronic acid and α‐l‐guluronic acid determines the properties of the alginate hydrogel. A higher percentage of α‐l‐guluronic blocks results in the formation of more rigid and brittle gels, while higher amounts of mannuronic acid blocks result in the formation of less rigid and more flexible gels (Ching et al. 2017). Additionally, the synergy achieved by combining alginate with prebiotics provides improved protection for probiotics within food systems, facilitated by their symbiotic relationship. This enhancement is attributed to prebiotics' ability to form three‐dimensional networks of microcrystals, which interact to form small aggregates. These aggregates contribute to better protection of probiotic cells in co‐encapsulation (Vivek et al. 2023). Alginate serves as an encapsulating material in various co‐encapsulation applications owing to its hydrophilic nature, ease of manipulation, and innocuousness. Moreover, alginate offers functionalities such as gelling, stabilizing, and thickening, which are highly desirable in microencapsulation. Alginate hydrogels are extensively used in probiotic cell co‐encapsulation, with calcium as the crosslinking agent to form calcium alginate. This is due to the non‐toxicity of Ca^2+^ compared to other cations, its biocompatibility, and low cost (Motalebi Moghanjougi et al. 2021; Parente et al. 2022). Microparticles of calcium alginate are commonly synthesized through two primary methods: extrusion and emulsification. In the extrusion method, a solution of sodium alginate is dripped into a solution containing a calcium salt. This process induces external ionic gelation, wherein the calcium ions in the solution react with the alginate to form a gel network (Liu et al. 2017). Alternatively, the emulsification method involves the internal ionic gelation of alginate within a water/oil emulsion. Here, alginate forms microparticles within the emulsion, facilitated by the presence of Ca^2+^. In both methods, gelation occurs when blocks of α‐l‐guluronic acid α‐l‐guluronic (G) within one alginate molecule become physically linked to blocks of α‐l‐guluronic acid α‐l‐guluronic (G) from another alginate molecule by Ca^2+^. This connection creates junction zones, resembling an “egg box” structure, which contribute to the formation of the gel network (Liu et al. 2017). Even though alginate is commonly used in microencapsulation and co‐encapsulation, there are some drawbacks, such as alginate microcapsules exhibiting high sensitivity to low pH and possessing a highly porous nature, making them susceptible to degradation under stomach conditions (Rodrigues et al. 2018). Nevertheless, the drawbacks can be overcome by mixing alginates with other polymer compounds to carry out structural modifications aimed at improving particle properties (Rodrigues et al. 2018). For instance, alginates have been combined with natural plant‐derived polysaccharides to enhance efficacy in encapsulating probiotics (Rodrigues et al. 2018). Research conducted showed that combining alginate with starch enhances the efficacy of various bacterial cells, particularly lactic acid‐producing bacteria (Homayouni et al. 2008). This improvement is attributed to the production of granules with a favorable prebiotic structure and effects within the microcapsules. It is integrated both capsule enhancement techniques to investigate the impact of galacto‐oligosaccharides and insulin on the survival of microencapsulated *Lactobacillus acidophilus* and *Lactobacillus casei* within calcium alginate capsules coated with chitosan in a simulated digestive system (Krasaekoopt and Watcharapoka 2014). Another strategy is applying a coating for microcapsules. Polycations like chitosan, poly‐amino acids, and whey proteins can be combined with alginate to decrease the porosity of the gel to achieve robust complexes that exhibit stability even in the presence of chelating agents like Ca^2+^. Their association with calcium alginate results in the creation of more stable capsules, enabling the formation of a double wall in the microcapsule structure (Liu et al. 2017). However, alginate has limitations in co‐encapsulation, including heat sensitivity, porosity, and weak barrier properties due to high molecular mobility and weak intermolecular interactions. These disadvantages can lead to premature release and reduced protection of encapsulated probiotics, necessitating modifications such as multilayer coatings or ionic crosslinking to improve its functionality (Liu et al. 2017).

##### 6.2.1.2 Chitosan

Chitosan is a linear cationic polysaccharide composed of d‐glucosamine and N‐acetyl‐glucosamine. It is polymerized by cross‐links formed in the presence of anions and polyanions. Commercially, chitosan is extracted using chitin, which is extracted from the shells of crustaceans by partial deacetylation (Thambiliyagodage et al. 2023). Chitosan is widely used in co‐encapsulation to coat sodium alginate beds, enhancing the viability of probiotics such as *Lactobacillus plantarum, Bifidobacterium lactis*, and *Lactobacillus acidophilus*. Chitosan's suitability as a coating material, rather than an encapsulating material, makes it a preferred choice in this application (Jantarathin et al. 2017; Mortazavian et al. 2008; Zaeim et al. 2019). It is noted that in acidic environments, the amine groups in chitosan, which carry a positive charge, can engage in electrostatic interactions with anionic polymers such as alginate (Qi et al. 2020). Further, chitosan has been shown to provide higher protection in co‐encapsulation, thereby enhancing the viability of *Bifidobacterium longum* in gastrointestinal fluid and high‐temperature conditions (Ji et al. 2019). The utilization of chitosan as an encapsulation agent for probiotic bacteria may have drawbacks due to its inhibitory properties against microorganisms, including lactic acid bacteria (Goy et al. 2009). However, studies have demonstrated its effectiveness in coating alginate beads containing probiotic bacteria, such as *Lactobacillus casei* and prebiotic inulin. For instance, chitosan‐coated microcapsules exhibited minimal reduction in cell count (0.7–0.9 log CFU/g) after exposure to simulated gastric conditions (Darjani et al. 2016). One finding indicated that chitosan coating significantly reduced the release of inulin from co‐encapsules, as 2% of inulin compared to uncoated co‐encapsules, which released five times more, resulting in a 10% loss of inulin (Zaeim et al. 2019). Chitosan coating improves the thermal stability of probiotic encapsulation systems, particularly when combined with microcrystalline cellulose, alginate, or xanthan gum. A 0.5% chitosan outer layer enhances the resilience of encapsulated *L. sporogenes*, ensuring better survival during baking at 90°C for 15 min. This protective effect makes chitosan a valuable component in probiotic delivery systems, especially for heat‐processed food applications (Mirzamani et al. 2021). Furthermore, it has proved the ability of chitosan as a coating wall material in microcapsules to preserve the probiotics from adverse conditions (Krasaekoopt and Watcharapoka 2014; Thambiliyagodage et al. 2023; Zaeim et al. 2019).

##### 6.2.1.3 Pectin

Pectin, an anionic polysaccharide derived from plant cell walls, is mainly comprised of lengthy chains of linear homogalacturonan that contain α‐(1, 4)‐linked galacturonic acids, rhamnogalacturonan I, and rhamnogalacturonan II. Galacturonic acids undergo methyl esterification to form either high methoxyl pectin or low methoxyl pectin (Yang et al. 2018). As in alginate, pectin can produce hydrogels when exposed to divalent cations like calcium, especially when methyl‐esterified to a level of 25%–50% (Cui et al. 2017). Even though the process of encapsulating probiotic bacteria in pectin follows a similar approach to that of alginate, studies have revealed that pectin‐based beads appear to be more resistant to gastric and intestinal conditions than their alginate‐based counterparts (Voo et al. 2011). Pectin beads outperformed alginate beads in terms of encapsulation efficiency, hardness, and resilience (Sandoval‐Castilla et al. 2010). This may be attributed to the slight shift in the arrangement of chains relative to each other, forming different crosslinking structures associated with Ca^2+^, which is referred to as the “shifted egg‐box” model (Voo et al. 2011). The effectiveness of pectin and alginate as encapsulating wall materials to protect and release *Lactobacillus bulgaricus* within the intestine was investigated (Hu et al. 2021). Extrusion was performed by releasing a mixed solution of pectin and alginate with probiotics into a crosslinking solution of CaCl_2_ (0.3% w/v) through a needle (30 mm in diameter). The findings of the study demonstrated that the formed microspheres were within the 140–156 μm diameter range with an 85.67% encapsulation efficiency. Further, the microcapsules exhibited moderate retention of probiotics after exposure to the simulation of saliva, gastric juice, and intestinal juice. *L. bulgaricus* was encapsulated with different prebiotics using pectin‐rice bran capsules prepared by ionotropic gelation (Chotiko and Sathivel 2016). In this study, all the treatments, including coated and uncoated probiotics, showed higher encapsulation efficiencies of more than 95%, attributed to the ability of pectin to entrap cells. *Lactobacillus acidophilus* LA‐5 was co‐encapsulated with different prebiotics, such as inulin, resistant starch, and rice bran, using external ionic gelation, optimizing the best treatment of high encapsulation yield of microcapsules with rice bran and inulin, as 91.24% and 90.59%, respectively (Raddatz et al. 2020). Another advantage of using pectin in co‐encapsulation is its ability to be utilized in the formulation of complex wall materials due to its wide compatibility with other wall materials. Another study highlighted the ability of pectin to be applied in complex wall materials to enhance the survivability of *L. rhamnosus* ZFM231; applied whey protein and pectin double layer in microencapsulation showed a significant improvement in the survivability of *L*. *rhamnosus* after exposure to simulated intestinal fluid and storage (4°C for 28 days and 25°C for 14 days) (Chen et al. 2023). Pectin‐based beads face challenges in probiotic encapsulation due to several reasons, such as their high solubility in water, which can lead to rapid probiotic release. Furthermore, its porous structure, low thermal stability, and weak mechanical properties limit effectiveness in applications requiring prolonged stability. Additionally, the gelation process of pectin results in increased sucrose content, making it less suitable for diabetic‐friendly formulations (Noreen et al. 2017).

##### 6.2.1.4 Carageenan

Carrageenan is commonly used as a natural food additive and thickener in food systems, extracted from marine macroalgae. It is a negatively charged linear polysaccharide comprised of disaccharide units of d‐galactose and 3,6‐anhydro‐d‐galactose (Sun et al. 2015). Among the three types of carrageenan, *κ*‐carrageenan and *λ*‐carrageenan are widely utilized for food, pharmaceutical, and commercial applications (Fauzi et al. 2020). The carrageenan is preferable for preparing probiotic beds due to its ability to form a 3D structured gel with metal ions. Since gel forms at low temperatures, probiotics are added to a dissolved carrageenan solution, which is prepared by heating around 60°C–80°C in cool conditions. Carrageenan, often combined with carrageenan and other polymers such as carboxymethyl cellulose, is utilized in microencapsulation and co‐encapsulation processes (Dafe et al. 2017; Gutiérrez‐Zamorano et al. 2019). One advantage of using carrageenan in co‐encapsulation is its ability to enhance stability and electronegativity in acidic conditions, thus safeguarding encapsulated probiotics, prebiotics, and other bioactive compounds compared to pectin and alginate (Gu et al. 2021). *Lactobacillus casei* 01 was encapsulated with κ‐carrageenan and various wall materials and prebiotics like resistant starch, lactulose, and lacto‐sucrose by extrusion using a 0.7 mm needle into a sterile gel cross‐linking solution. High encapsulation efficiency and viability of probiotics were achieved by microcapsules formed using carrageenan. Previous studies have successfully co‐encapsulated *Bacillus coagulans* spores with vitamin B9 using a composite hydrogel made from κ‐carrageenan, chitosan, and gellan gum (Srivastava et al. 2023; Ta et al. 2021). The co‐encapsulated matrix demonstrated effectiveness in preserving the integrity of both co‐encapsulated folic acid and bacterial spores within the acidic gastric environment, ensuring their viability and stability. Additionally, locust bean gum was used to enhance the gelling properties of carrageenan in the encapsulation of *Lactobacillus bulgaricus* using the extrusion method. This study found that more than 8 log CFU/g of the viability of encapsulated *L. bulgaricus* was maintained after 2 h of incubation under simulated gastric fluid (Shi et al. 2013). The ability of carrageenan, particularly κ‐carrage, to facilitate the transition of cellular components into a glassy state, which is characterized by a solid, non‐crystalline structure, inhibits the formation of ice crystals within cells. Therefore, carrageenan can act as a cryoprotectant, and it is beneficial in co‐encapsulation since probiotics are preservedfreeze‐drying process. The incorporation of κ‐carrageenan helps to protect the cellular integrity and functionality of *S. thermophilus* by minimizing damage caused by ice crystal formation, freeze‐drying, and the process was able to achieve encapsulation efficiency of 87.814% (Yashaswini et al. 2024). de la Cruz Pech‐Canul et al. (2020) reported that probiotics are released at a slower rate from carrageenan‐based hydrogels compared to alginate‐based hydrogels.

##### 6.2.1.5 Starch and Resistant Starch

Starch is a polysaccharide with glycosidic linkages connecting glucose molecules, and it consists of two main components: amylose, a linear polymer with d‐glucopyranose residues linked by α‐1‐4 glycosidic bonds, and amylopectin, a branched glucose polymer joined by both α‐1‐4 and α‐1‐6 glycosidic links, which facilitate branching (Junejo et al. 2022). The removal of water from hydrogel capsules during drying causes them to collapse, resulting in a reduction of sphericity and a shift from spherical to irregular shapes. The addition of a filler agent, such as starch, in co‐encapsulation helps maintain microcapsule shape during drying by offering structural support and protecting the capsules from collapse and shrinkage (Chan et al. 2011).

Certain drawbacks of normal starch can be overcome by various chemical and physical modifications. An emerging trend involves exploring resistant starch as a wall material in co‐encapsulation, driven by its advantageous properties in encapsulation such as thermostability, enhanced protection for probiotics, and the capacity to create innovative combinations of wall materials, ultimately leading to the improvement of encapsulation efficiency in prebiotic/probiotic (symbiotic) combinations for microencapsulation (Muhammad et al. 2021). The incorporation of resistant starch (RS) in co‐encapsulation processes plays a crucial role in maintaining the thermal stability of prebiotics and probiotics, such as during spray drying. This inclusion not only prolongs the shelf life of microcapsules but also enhances targeted delivery mechanisms. Specifically, RS facilitates improved enteric delivery by promoting probiotic cell adherence to starch granules and the subsequent release of bacterial cells within the large intestine (Heidebach et al. 2009). Resistant starch's resistance to digestion mainly depends on the percentage of amylose content of starch. Consequently, lower digestibility and more resistance are given by a high amount of amylose content (Li et al. 2023). Further, RS is categorized into 5 groups based on the resistant mechanism and starch structural composition (RS I, II, III, IV, and V). Therefore, RS lets one choose a more suitable type for co‐encapsulation as a wall material with different properties. For instance, RS1 is enzyme‐binding limited, RS2 lacks surface pores hindering enzyme binding, RS3 prevents the formation of retrograded structures, and RS4 contains chemical crosslinks that delay enzyme binding. RS5, a thermally stable starch, forms a resistant amylose‐lipid complex that resists α‐amylase enzyme hydrolysis. This mechanical and functional understanding is important to apply RS in designing proper wall material combinations with improved protection to the symbiotic core and resistance to passage through the digestive tract (Junejo et al. 2022).

A study reported that the incorporation of RS from maize and potato has significantly enhanced the encapsulation efficiency of microcapsules with *L. acidophilus* at 95.80% and 94.30%, respectively (Muhammad et al. 2021). Notably, the addition of maize‐resistant starch showed the highest survivability of *L. acidophilus* after exposure to gastric conditions, which is attributed to its high thermal and chemical resistance. Previous research studies reported superior stability and survival of probiotics like *Bifidobacterium adolescentis* ATCC 15703 and *Lactobacillus casei* ATCC 39392 in high‐amylose corn‐resistant starch compared to wheat and rice starch (Zanjani et al. 2018).

In addition to these polysaccharides, there are modified cellulose, modified starch, and novel polymers used in probiotic co‐encapsulation. Modified cellulose was used to microencapsulate *Lactobacillus acidophilus* (La‐05) and *Bifidobacterium lactis* (Bb‐12) by spray drying of a carrier solution containing cellulose acetate phthalate as wall material and incorporated FOS as the prebiotic (Fávaro‐Trindade and Grosso 2002). The results revealed that *B. lactis* was more suitable to be encapsulated by the spray drying process under inlet and outlet temperatures of 130°C and 75°C than *L. acidophilus*. It is reported that cellulose esters, including microcapsules prepared by spray drying, showed a higher stability against digestive conditions (Gilley et al. 2017). This may be due to the insolubility of microcapsules in acid media (pH ≤ 5) and solubility in alkaline pH (≥ 6). Therefore, microcapsules formulated with cellulose acetate phthalate can protect the encapsulated core material against acidic gastric conditions and pass through the stomach, gradually releasing the core material in the slightly neutral to alkaline conditions of the intestine.

Carboxymethyl cellulose was combined with κ‐carrageenan for microencapsulation of *Lactobacillus plantarum* ATCC:13643 to increase survival and protection in the gastrointestinal tract and bile salt solution. Microencapsulation of probiotics was performed by the extrusion method. External gelation of CMC and κ‐carrageenan was conducted and mixed with probiotics, then extruded to CaCl_2_/KCl cross‐linking solution (Dafe et al. 2017). The utilized mucilage, an exopolysaccharide produced by bacteria and fungi, was used in the preparation of alginate‐based edible coating with *Lactobacillus casei* LC‐01 (Rodrigues et al. 2018). Different kinds of gums, primarily microbial polymers, are used in the food industry as texture enhancers, thickeners, and encapsulating wall materials. There is a potential to develop new wall materials and combinations suitable for the co‐encapsulation of prebiotics and probiotics. The effectiveness of various gums has been studied on microencapsulating probiotics as a wall material (Jiménez‐Pranteda et al. 2012). Xanthan gum, gellan gum, pullulan gum, and jamilan were tested as wall materials for encapsulating *Lactobacillus plantarum* CRL 1815 and *Lactobacillus rhamnosus* ATCC 53103 through the extrusion method using an electrostatic droplet generator. The wall material mixture of xanthan gum: gellan gum (1%:0.75%) exhibited superior protection for the encapsulated core materials, *L. plantarum* and *L. rhamnosus* , against simulated digestive conditions (Jiménez‐Pranteda et al. 2012). In addition to polysaccharides, various proteins have also been used in co‐encapsulation, either as a single material or combined with polysaccharides. Milk proteins, gelatin, and plant‐derived proteins are commonly utilized in probiotic‐prebiotic co‐encapsulation due to their advantageous effects.

#### 6.2.2 Proteins

The utilization of proteins in encapsulation has been widely observed, primarily due to their favorable functional characteristics. In contrast, specific proteins exhibit a relatively high cost and possess the potential to elicit allergic reactions. However, it is important to highlight that plant‐based proteins are widely recognized for their reduced allergenic potential in comparison to proteins sourced from animals (Desai and Park 2005). The process of protein denaturation has the potential to enhance the mechanical properties of proteins, thereby increasing their effectiveness in providing protection. Denaturation of proteins leads to the aggregation of protein molecules, which subsequently form a matrix that is both more durable and flexible but remains insoluble. The enhanced characteristics of denatured proteins effectively restrict the release and exposure of cells within a simulated gastric environment (Rajam et al. 2012). Encapsulated probiotics are released by breaking down the outer encapsulating protein wall as a result of digestion with the presence of pepsin in human gastric digestion (Kent and Doherty 2014).

##### 6.2.2.1 Gelatin

Gelatin is a water‐soluble polymer produced by partially hydrolyzing natural collagen, which is the main element of the connective tissue's extracellular matrix. It is made up of a variety of polypeptide chains of different lengths. Gelatin exhibits solubility in water when subjected to temperatures above 40°C, resulting in the formation of a transparent and thick fluid. In contrast, the substance undergoes a transition into a gel‐like state when exposed to temperatures lower than the ambient environment, like the use of transglutaminase. Gelatin has been reported to enhance the survivability of encapsulated *Saccharomyces boulardii* in simulated gastric conditions (Arslan et al. 2015; Nawong et al. 2016). Gelatin's amphoteric nature allows it to form strong complexes with anionic polymers, creating durable, oxygen‐impermeable capsules. Its linear structure enhances oxygen barrier properties, while interactions with polysaccharides improve resistance to cracking and breaking (Koh et al. 2022).

##### 6.2.2.2 Milk Proteins

Milk proteins are a natural delivery mechanism for probiotic bacteria due to their structural and physico‐chemical characteristics. Proteins' excellent gelation characteristics are important in microencapsulation and co‐encapsulation of probiotics and prebiotics. Findings of the previous investigations are encouraging, and utilizing milk proteins as a novel approach with higher biocompatibility (Heidebach et al. 2009). Whey protein and casein are the most commonly used milk proteins in the co‐encapsulation of probiotics and prebiotics (Heidebach et al. 2010).

##### 6.2.2.3 Casein

Casein is a milk protein used in encapsulating different probiotics such as *Lactobacillus* F19 and *Bifidobacterium* Bb12. A study showed that both encapsulated probiotics recorded higher survival compared to non‐encapsulated ones after exposure to low pH and simulated gastric conditions. The improved survival of encapsulated probiotic cells is attributed to the buffering capacity of casein, which has protected cells under low pH conditions (Heidebach et al. 2010). Furthermore, casein is suitable for preparing probiotics containing microcapsules due to its gelation ability and thermal resilience (Heidebach et al. 2009). Sodium caseinate is a widely used encapsulating material for probiotics due to its amphiphilic nature, strong emulsifying and gelling properties, and high thermal stability. Its affordability, biocompatibility, and ability to form protective gel beads make it advantageous for encapsulation. However, its use is limited by potential allergenicity and immunogenicity, particularly for individuals with dairy allergies (Koh et al. 2022).

##### 6.2.2.4 Whey Protein

Whey protein is a combination of globular proteins extracted from whey, with two main components being β‐lactoglobulin and α‐lactoalbumin. Whey proteins can form gels with mild heat treatment without using any chemicals as a cross‐linking agent. Therefore, it can be heat‐treated and pre‐denatured at 35°C to 40°C when encapsulating probiotics, as high temperatures are not suitable for probiotic bacteria and frequently cause cell degradation. Whey protein acts as a shield for probiotics against the stomach's acidic environment, and protein helps to minimize probiotic cell losses during drying (Doherty et al. 2015; Soukoulis et al. 2017). *Lactococcus lactis* subsp. lactis R7 was co‐encapsulated with inulin as a prebiotic using whey protein by spray drying at 100°C of inlet temperature, an outlet temperature of 68°C, and a drying air flow rate of 3.00 m^3^/min. Microcapsules formed with whey protein exhibited robust viability at 13.0 log CFU/g and a high encapsulation yield of 94.61%. The viable cell counts remained stable, exceeding 8.0 log CFU/g during a six‐month storage period at various temperatures. The use of whey protein in spray drying effectively protected *L. lactis* R7 through simulated gastrointestinal tract passage and demonstrated thermal resistance at the applied temperatures (Rosolen et al. 2019). Additionally, *Lactobacillus rhamnosus* ATCC 7469 was encapsulated using inulin as a prebiotic by freeze drying method. Crystalline nanocellulose and whey protein were used as wall materials (Maleki et al. 2020). The highest encapsulation efficiency (89.60%) and survival of probiotic cells were achieved with a microencapsulation composition of whey protein: crystalline nanocellulose: inulin as 57.22%: 25.00%, 17.78% respectively. Whey protein serves as a protectant for microorganisms during freeze drying by accumulating within the cells, reducing the osmotic difference between the internal and external environments (Chotiko and Sathivel 2016; Meng et al. 2021). Whey proteins serve as an effective medium for probiotic encapsulation due to their high nutritional value, amphoteric nature, and superior gelation, thermal stability, hydration, and emulsification properties. Their resistance to pepsin digestion enhances probiotic protection, while their ability to form complexes with polysaccharides like gum Arabic, maltodextrin, and pectin improves encapsulation efficiency. Heat‐induced denaturation further strengthens their structural integrity, making them suitable for preserving probiotics in various applications (Koh et al. 2022).

##### 6.2.2.5 Plant Protein

The food, cosmetics, and pharmaceutical industries are adhering to the environmentally conscious trend. Zein, a hydrophobic protein derived from maize gluten, is widely recognized as a prominent plant protein employed in encapsulation applications. It is insoluble in water while displaying solubility in aqueous alcohols, glycols, and acetone. The substance is abundantly available, biodegradable, and thermally stable, and it also acts as an effective oxygen and aroma barrier. These attributes make it highly advantageous for microencapsulation techniques in the food sector (Kasaai 2018). Globulin protein extracted from peas has been investigated as a potential encapsulating agent for the microencapsulation of probiotics in the food industry (Lam et al. 2018). Pea protein isolate has demonstrated higher survival rates of probiotics such as *Lactobacillus reuteri* ATCC 53608 in simulated gastric fluid (Wang et al. 2007). Soy protein isolate, consisting of glycinin and β‐conglycinin, is appropriate for the microencapsulation of probiotics (González‐Ferrero et al. 2018). Encapsulation using soy proteins via the spray drying method achieved higher survival rates of *Lactobacillus casei* in simulated gastric conditions and extended shelf life with better retention of *Lactobacillus casei* (Hadzieva et al. 2017). Zein, soy, and other plant‐based proteins are effective coating materials for probiotic encapsulation due to their amphiphilic nature, biocompatibility, biodegradability, water insolubility, and high resistance to gastric juice. These properties enhance the stability and protection of encapsulated probiotics. However, their application in large‐scale production is limited by their instability, tendency to aggregate in aqueous solutions, and heat‐induced gel formation, which can affect processing efficiency and consistency. Table 1 shows an overview of probiotic and prebiotic co‐encapsulation using different encapsulation methods and different wall and core materials (Koh et al. 2022; de la Cruz Pech‐Canul et al. 2020). Table 1 shows an overview of probiotic and prebiotic co‐encapsulation using different encapsulation methods and different wall and core materials.

**TABLE 1:** An overview of probiotic and prebiotic co‐encapsulation using different encapsulation materials and techniques.

Wall material	Core materials		Co‐encapsulation technique	Highlights	References
Alginate	Prebiotic(s)	Probiotic(s)	Extrusion by external ionic gelation Composition of feed solution: 2% sodium alginate, 10% prebiotic, and 100 mL sterile distilled water Hardening: CaCl_2_, Nozzle size: 0.3 mm Air pressure: 2.72 kg/cm^2^ Distance between nozzle and hardening solution: 30 cm	The highest Encapsulation Efficiency (EE) was recorded by inulin, used as an encapsulating matrix (96.75%), which was the only stable treatment with viable probiotics for 120 days at −18°C, at 25°C	Poletto et al. (2019)
	Inulin Rice bran Resistant starch	*Lactobacillus acidophilus*			
Alginate Chitosan	Long chain inulin Resistant starch (high amylose resistant granules)	*Lactobacillus plantarum* ATCC 8014 *Bifidobacterium lactis*	Microparticles prepared by electro‐hydrodynamic atomization Coating of alginate, probiotic, and prebiotic mixture with chitosan using electro spray Needle size: 0.4 mm Voltage: 9.5 kV Flow rate: 5 mL/h Tip‐to‐collector distance: 1 cm Chitosan coating time: 30 min Recovery of microcapsules: freeze drying at −56°C and 100 mTorr for 48 h	The highest Encapsulation Yield (EY) was obtained by the resistant starch added treatment (99.2%) Lower wrinkles on microcapsules were observed by adding inulin or starch as compared to microcapsules without prebiotics Both the addition of prebiotics and the encapsulation process increased the viability of both probiotics under Stimulated Gastrointestinal Conditions (SGI) conditions Co‐encapsulated *L. plantarum* with inulin showed higher viability and lower reduction of cells (lost 3.4 log cycles) compared to free cells (lost 6.5 5 log cycles) after exposure to SGI Co‐encapsulated *L. plantarum* with resistant starch showed higher survival and lower reduction than the free one, with 3 3‐log cycle viability loss Similar trends were observed between the encapsulated and free treatments of *B. lactis* with inulin and resistant starch Survival of *B. lactis* in encapsulated treatments with inulin showed 7.01 and 6.51 CFU/g at −18°C and 4°C after 90 days Survival of *B. lactis* in encapsulated treatments with resistant starch showed 7.05 and 5.92 CFU/g at −18°C and 4°C after 90 days Survival of co‐encapsulated *L. plantarum* with resistant starch was 1 CFU/g at 25°C after 90 days	Zaeim et al. (2019)
Alginate Chitosan	Inulin Jerusalem artichoke	*Lactobacillus acidophilus* TISTR 1338	Extrusion and drying by freeze drying Needle size: 30 G Hardening: 0.1 M CaCl_2_, 30 min Distance between nozzle and hardening solution, cm Recovery of microcapsules: freeze drying at −46°C for 36 h	Chitosan double‐coating improved the viability of encapsulated cells The highest EE (89.93%) was observed in inulin‐added microcapsules, and the highest survival rate was obtained by Jerusalem artichoke‐added microcapsules (85.97%) 3% prebiotic with 3% alginate and 0.8% chitosan was the best combination with the highest survival of microcapsules Jerusalem artichoke added microcapsules showed a superior viability of probiotics after heat treatment of 70°C for 30 min compared to control and inulin added microcapsules	Jantarathin et al. (2017)
Carboxymethyl Cellulose Chitosan	Cyclodextrins (*α*, *β*, ∞) Hydroxypropyl Methylcellulose (HPMC) Hydroxypropyl‐β‐Cyclodextrins	*Lactobacillus rhamnosus* GG LMG 18243	Microparticles Microparticles were formed by adding carboxymethyl cellulose aqueous solutions into the chitosan solution through a syringe Needle size: 0.7 mm Aging: 30 min	The cyclodextrin‐added treatments improved the EE and viability compared to the cyclodextrin‐free treatments The viability of the cyclodextrin‐added samples was high after 30 days of storage at room temperature	Singh et al. (2018)
Sweet whey	Inulin Polydextrose	*Bifidobacterium BB‐12*	Spray drying Air inlet temperature: 150°C Outlet temperature: 50°C ± 3°C	EE was varied as SW > SWI > SWP as 95.43 > 93.55 > 88.30, respectively. (SW: microcapsules with sweet whey; SWI: microcapsules with sweet whey and inulin; SWP: microcapsules with sweet whey and polydextrose) Whole sweet whey added microcapsules demonstrated the highest survival rate compared to prebiotics added treatments after exposure to SGI All the microcapsules showed a high count of *bifidobacteria*, low moisture content, and low water activity Heat stability and the survival of probiotics: The most stable treatment under 70°C for 10 min was SW	Pinto et al. (2015)
Full‐fat goat milk	Inulin Oligofructose	*Bifidobacterium* BB‐12	Spray drying Air inlet temperature: 150°C ± 1°C Outlet temperature was 50°C ± 3°C Flow rate: 20 mL/min Airflow rate: 35 m^3^/h Compressor air pressure: 0.7 MPa Feed solutions were formulated with sterile distilled water, homogenized and heated at 80°C ± 2°C for 30 min	All treatments improved the survival of the probiotics after thermal treatments Microcapsules prepared only with full‐fat goat milk recorded the highest EY as 97.43% The significantly highest survival of probiotics was recorded by goat milk (9.83 CFU/g) after spray drying and exposure to SGI conditions (94.29%)	Verruck et al. (2017)
Pea protein isolate Alginate	Fructo‐oligosaccharides (FOS)	*Bifidobacterium adolescentis*	Extrusion Needle: gauge 20/27 Hardening: CaCl_2_ solution, 30 min	The addition of FOS gives a higher protection to the encapsulated *B. adolescentis* throughout processing and when exposed to SGI *B. adolescentis* cells entrapped within capsules are protected from acidic gastric juice over a 2 h period The size of microcapsules was affected by the needle gauge, protein level, and prebiotic level	Klemmer et al. (2011)
Alginate Fenugreek gum Locust bean gum	Fructo‐oligosaccharides (FOS) Lactulose Maltodextrin	*Pediococcus pentosaceus* KID7 *Lactobacillus plantarum* KII2 *Lactobacillus fermentum* KLAB6 *Lactobacillus helveticus* KII13	Extrusion‐ionotropic gelation Nozzle size: gauge 26 Flow rate: 2.2 mL/min Hardening: 0.05 M CaCl_2_, 30 min	All four probiotics showed higher EE of more than 95%, and the sizes of microcapsules were within a range of 25–520 μm Survival of probiotics after encapsulation was varied as *L. helveticus* < *P. pentosaceus* < *L. fermentum* < *L.plantarum* as 8.95 < 8.56 < 8.18 < 8.11 Log_10_ CFU/g The highest survival rate after exposure to SGI conditions was observed in *L. fermentum* as 96.44% The highest survival rate after exposure to colonic fluid was observed in *L. plantarum* as 97.7% Freeze‐dried bacterial cells survived for 3 months at 4°C as KID7, KLAB6KII2, and KII13 retained a relative viability of 92.35%, 92.19%, 90.26%, and 86.35%, respectively	Damodharan et al. (2017)
Alginate	Arabinoxylan extracted from maize	*Latobacillus plantarum*	Microspheres formed by co‐gelation of alginate using a digital gear drive pump and Freeze drying Pump speed: 50 rpm Gelation time: 30–60 min	Incorporation of prebiotic arabinoxylan increased the EE, bile salt resistance, and gastric stability	Wu and Zhang (2018)
Alginate	Inulin Cactus pear powder Apple marc powder	*Lactobacillus plantarum* UAM17 *Enterococcus faecium* UAM18 *Aerococcus viridans* UAM21b *Pediococcus pentosaceus* UAM22a	Microcapsules prepared by emulsification, internal ionotropic gelation of sodium alginate Mixed solution of probiotic and probiotics with maize oil and tween 80 was homogenized using magnetic stirring for 10 min at 400 rpm to form the emulsion, and acetic acid and further maize oil were added during stirring in order to form the micro‐beads Hardening time: 20 min	*E. faecium* UAM18 showed significantly higher EY, followed by *P. pentosaceus* UAM22a The highest EY for each four strains was obtained by adding apple marc powder added microcapsules Cactus pear powder added microcapsules showed a lower particle size compared to inulin and apple marc powder added ones *A. viridans* showed the highest acid tolerance, followed by *E. faecium* The highest cell viability was recorded in all strains, encapsulated with inulin and *L. plantarum* and *P. pentosaceus* showed higher viability after 30 days of storage	Serrano‐casas et al. (2017)
Palm and palm kernel oil	Inulin Polydextrose	*Lactobacillus acidophilus* (LAC‐04)	Solid lipid microparticles were prepared by spray chilling the solution The solution, composed of probiotic and lipid, either with prebiotic or without prebiotic, was homogenized at 7000 rpm for 1 min The mixture was atomised using a spray chiller comprising a double fluid atomizer (with a size of 1.2 mm) cold chamber at 15°C, and under a pressure of 5 bar	The inclusion of inulin or polydextrose did not affect the morphology of the solid lipid microparticles All produced microcapsules significantly enhanced the survival rate of *L. acidophilus* Solid lipid microparticles produced with polydextrose reported the highest viability of *L. acidophilus* for 120 days at −18°C, 7°C, and 22°C	Okuro et al. (2013)
Milk protein Alginate	Inulin	*Bifidobacterium animalis* subsp. *lactis* Bb12	Emulsion method and extrusion method *Emulsion method* Emulsion: homogenization of the mixture of skimmed milk with sunflower oil with 0.5% (w/w) soya lecithin Recovery of microcapsules: centrifugation Speed: 6000 rpm Temperature: 4°C Time: 2 min *Extrusion method* 1 g of probiotic culture was added to 10 mL of 1% (w/v) sodium alginate solution and mixed. Hardnening: 0.1 M CaCl_2_, 30 min Needle size: 0.5 mm	The size of microcapsules prepared by protein and alginate containing only Bb12 was 204 ± 18 μm and 1.7 ± 0.1 mm, respectively Both emulsion and extrusion encapsulation methods protected probiotic cells and provided higher resistance to the GSI conditions compared to free cells Co‐encapsulation of Bb12 in a protein matrix with 1% w/w inulin and 0.5% w/w ascorbic acid was more efficient up to 6 weeks of storage	Kumherová et al. (2020)
Alginate	Oligosaccharides Including Galacto‐oligosaccharides Isomalto‐oligosaccharides Fructo‐oligosaccharides (FOS) Xylo‐oligosaccharides	*Lactobacillus fermentum* L7	Extrusion Alginate‐based polymeric solution, added with probiotics and prebiotics, was dropwise added to the hardening solution using a syringe Needle size: 0.45 mm Hardening: 2% (w/v) CaCl_2_ solution, 30 min	The EY of *L. fermentum* co‐encapsulated with oligosaccharides was in the range of 79.52%–89.75% The significantly highest EY was recorded by FOS co‐encapsulated cells as 89.75 ± 0.6 The size of microcapsules was not significantly varied with prebiotics and within a range of 2.34 ± 0.27–2.51 ± 0.03 mm The highest survival of *L. fermentum* L7 cells was recorded by microcapsules prepared by FOS as 10 ± 0.05 log CFU/g after 2 h exposure to simulated gastric juice. *L. fermentum* L7 co‐enapsulated with FOS showed the highest viability after exposure to simulated intestinal juice	Liao et al. (2018)
Alginate	Potato starch *Plantago psyllium* Inulin	*Lactobacillus casei* Shirota Two strains of *Lactobacillus plantarum* (Lp33 and Lp17)	Emulsification and alginate beads Emulsion was prepared by mixing prebiotics and probiotics in distilled water and emulsified with edible oil by stirring at 10,000 rpm for 15 min using a magnetic stirrer Sodium alginate was added to the mixture, and the emulsion was injected using a peristaltic pump Diameter of hose: 3 mm Needle size: 0.1 mm Hardening: CaCl_2_, 30 min Flowrate: 1 g/min Distance between nozzle and hardening solution:20 cm	The EE was significantly affected by the added prebiotic and probiotic types LP 17 showed the highest EE compared to the other two strains, with each prebiotic *P. psyllium* and inulin showed superior protection and prebiotic activity compared to potato starch Comparatively, the highest retention of (more than 70%) probiotics was recorded by *P. psyllium* added microcapsules after exposure to SGI conditions Co‐encapsulation process successfully protected LP33 and LP 17 strains with added prebiotics for 30 days of storage at 22°C	Peredo et al. (2016)

## 7 Applications of Probiotics and Prebiotics Co‐Encapsulation in the Functional Food Development in the Food Industry

**FIGURE 10:**
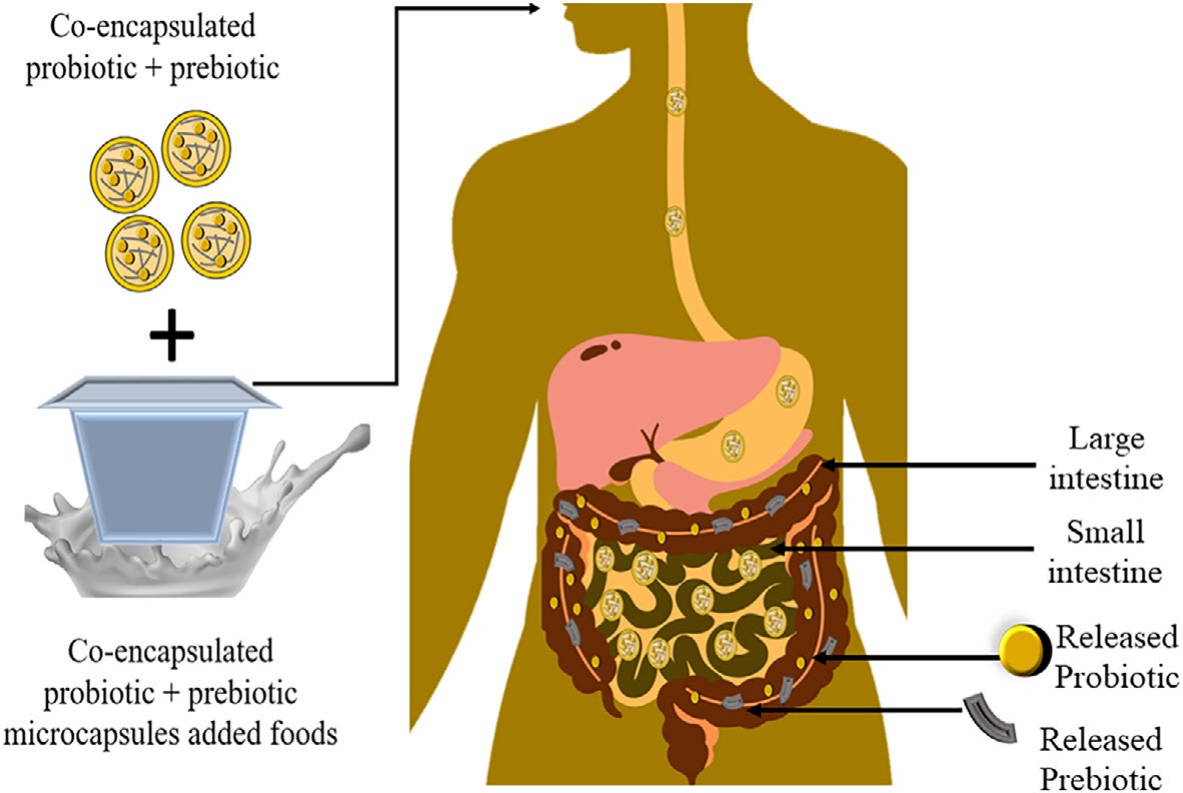
Fortification of foods using co‐encapsulated probiotics with prebiotics and their targeted delivery in the human digestive tract.

Co‐encapsulation has been currently used as a successful method to deliver probiotics and prebiotics to the targeted destination in the human digestive tract while keeping them active during the passage. Figure 10 shows the fortification of foods using co‐encapsulated probiotics with prebiotics and their targeted delivery in the human digestive tract.

Due to the advancements of various co‐encapsulation techniques, fortified food with prebiotics and probiotics has been used in the functional food industry as food supplements. Table 2 shows the different applications of co‐encapsulated probiotics and prebiotics in the food industry.

**TABLE 2:** Applications of co‐encapsulated probiotics and prebiotics in the food industry.

Functional food	Probiotic(s)	Prebiotic(s)	Co‐encapsulation technique	Highlights	References
Yoghurt	*Lactobacillus plantarum* TISTR1465	*Eleutherin Americana* Polysaccharide Commercial FOS	Extrusion Injected the mixed solution of cell suspension + sodium alginate solution + *E. americana* oligosaccharide extract through a syringe Needle size: 23 G Hardening: 0.1 M CaCl_2_ solution	The highest EE (93.52%) was recorded by alginate‐commercial FOS added microcapsules compared to *Eleutherin Americana* polysaccharide extract added treatment Alginate‐ *E. americana* oligosaccharide extract added microcapsules showed the highest survival of *L. plantarum* after exposure to SGI and showed the highest survival of *L. plantarum* in yoghurt as 8.45 ± 0.1 after 4 weeks of storage The yoghurt prepared with encapsulated cells showed less acidification than with free cells	Phoem et al. (2019)
Doogh (A typical Iranian drink made from fermented milk)	*Lactobacillus plantarum* LS5	*Helianthus tuberosus* inulin	Alginate beads Alginate, probiotic mixture, was added to canola oil and 0.5% Tween 80 The mixture was homogenized at 900 rpm for 20 min Calcium alginate beads were formed by adding a CaCl_2_ solution	The addition of *L. plantarum* LS5 (free or encapsulated) increased acid development in Doogh during storage *L. plantarum* encapsulated with 2% inulin showed the highest survival in doogh after 22 days of storage, as 8.12 ± 0.01 log CFU/mL Encapsulated probiotics added to doogh recorded the lowest phase separation compared to free probiotic bacteria added to doogh Sensory attributes of doogh were not significantly changed with the addition of free and encapsulated *L. plantarum* with or without inulin	Hashemi et al. (2014)
Reduced‐fat cream cheese	*Lactobacillus rhamnosus*	β‐glucan	Spray aerosol method Microbial suspension in alginate was added to the hardening solution using a pump Pump speed: 12 mL/min Air pressure: 500 kPa Solution was added to counter the aerosol of the CaCl_2_ solution Pressure: 400 kPa Flow rate: 9 mL/min	The addition of encapsulated and non‐encapsulated *L. rhamnosus* to cheese increased the buttery flavor (diacetyl compounds), and non‐encapsulated showed more effect compared to the encapsulated form	Ningtyas et al. (2019)
Gouda‐type cheese	*Bifidobacterium lactis*	β‐cyclodextrin Arabic gum	Spray drying Air inlet temperature: 160°C Air inlet flow rate: 5 mL/min	The highest yield and retention of probiotics were observed in microcapsules encapsulated with β‐cyclodextrin‐arabic gum after the spray drying process *B. lactis* and added prebiotics did not affect the physicochemical parameters of the Gouda‐type cheese Survival of microencapsulated *B. lactis* was 1.89 ± 0.25 × 10^11^ CFU/g in the probiotic powder and above 10^8^ CFU/g in the cheese after the ripening process of 40 days	Borrás‐Enríquez et al. (2018)
Soymilk	*Lactobacillus rhamnosus* GG	Resistant and waxy starches	External gelation method (Alginate‐gum microcapsules) A mixed alginate solution of probiotic, locust bean gum, and starch was added to the CaCl_2_ solution, atomization with air and feed flow rates of 140 L/h and 3 mL/min, respectively	Encapsulated *L. rhamnosus* showed better viability after incorporation into soy milk The addition of modified waxy starch increased the stability of microcapsules under stress conditions such as freeze drying, heat treatment, and during storage	Cheow et al. (2016)
Chocolate	*Lactobacillus acidophilus* (La5) *Lactobacillus rhamnosus* (LGG) *Lactobacillus sanfranciscensis* *Lactobacillus plantarum* *Lactobacillus casei 431* *Bifidobacterium animalis* subspp. lactis (Bb12) *Streptococcus thermophilus*	Cocoa powder FOS	Sodium alginate capsules and emulsion‐based freeze‐dried techniques The emulsion was prepared by homogenizing ingredients, including cocoa powder, sodium alginate, and FOS dissolved in Milli‐Q water for 15 min at 10,000 rpm Formed microcapsules were freeze‐dried at −50°C	The highest EE was achieved by microcapsules formed using cocoa powder + FOS, as 5.36% for *L. acidophilus* La5, and followed by cocoa powder added microcapsules as 93.40% for *L. casei* Higher viability of probiotics was maintained up to 180 days at 4°C by encapsulating with cocoa powder and FOS Co‐encapsulated probiotics showed higher survival in added chocolate for 90 days under storage at both 4°C and 25°C	Hossain et al. (2021)
Orange juice	*Lactococcus lactis* ABRIINW‐N19	FOS Inulin	Alginate microcapsules are formed by extrusion The homogeneous solution of probiotic, gum, and prebiotic was extruded Nozzle size: gauge 21 Hardening solution: CaCl_2_	The mean diameters for alginate, Persian gum mixed with FOS, and inulin were 350–430 and 460–560 μm All microcapsules showed higher EE than 98% The viability of probiotic cells in simulated digestive conditions was improved with the encapsulation and addition of prebiotics and gums The lowest reduction of probiotics was achieved by microcapsules prepared with 1.5% alginate +0.5% pectin gum +2% inulin when exposed to stimulated digestive conditions and during the 6‐week storage of fortified orange juice at 4°C All co‐encapsulated treatments showed high stability of viable cells for 6 weeks of storage in orange juice at 4°C	Nami et al. (2020)
Cupcake fortified with fresh microcapsules at a level of (5% w/w)	*Lactobacillus plantarum* ATCC8014	Maltodextrin	Emulsion method Probiotics were added to the alginate solution containing maltodextrin and/or pectin Emulsion was prepared by adding the aqueous phase to the canola oil and homogenized Alginate capsules were formed by adding CaCl_2_ and harvested by centrifugation	The maltodextrin and pectin added microcapsules strengthened the protection and the viability of *L. plantarum* exposed to SGI conditions Co‐encapsulated *L. plantarum* with pectin or maltodextrin has significantly increased the survival of the probiotic during the baking process compared to free samples The addition of encapsulated *L. plantarum* with maltodextrin, pectin did not affect the sensory attributes compared to the control cupcake	Dong et al. (2020)
Sausage	Thermotolerant lactic acid bacteria *Enterococcus faecium* UAM1 *Pediococcus pentosaceus* UAM2	Cactus pear peel flour Apple Marc flour Inulin	Microcapsules of sodium alginate and pectin mixture Probiotics were resuspended in CaCO_3_ solution containing sodium alginate, pectin, and prebiotics This aqueous phase was added to maize oil with an acetic acid mixture and homogenized at 400 rpm for 10 min to form the emulsion, and allowed to form micro‐gel beads	Textural properties were not notably changed due to the addition of microcapsules The addition of co‐encapsulated microcapsules increased the oxidative stability of lipids in sausage during storage Co‐encapsulated thermotolerant strains with cactus pear peel and apple marc peel flours safeguarded probiotics in sausage in storage	Barragan‐Martınez et al. (2019)
Yoghurt The fortification level of microencapsulated beads was 10% w/w	*Enterococcus durans* 39C	Inulin Fenugreek gel	Extrusion Alginate‐prebiotics mixture (inulin and/or fenugreek) was extruded through a nozzle (gauge 21) into CaCl_2_ solution	The addition of prebiotics increased the size of microcapsules Co‐encapsulated microcapsules with *E. durans* showed higher EE Co‐encapsulated *E. durans* with fenugreek showed the highest survival after exposure to simulated digestion conditions The addition of inulin and fenugreek slightly increased the acidity of yoghurt Survival of added *E. durans* in yoghurt was increased by co‐encapsulation with alginate‐psyllium blend with a fenugreek formulation	Haghshenas et al. (2015)
Ice cream	*Lactobacillus casei* (Lc‐01) *Bifidobacterium lactis* (Bb‐12)	Resistant starch	Emulsification An alginate mixture of probiotic and prebiotic was added to canola oil and emulsified at 400 rpm for 20 min. Calcium alginate beads were separated after adding the calcium solution	Probiotic survival was raised at a rate of 30% during the storage for 180 days at 20°C by encapsulation. The addition of co‐encapsulated microcapsule did not show any undesirable effects on the sensorial properties of the ice cream The loss of co‐encapsulated *L. casei* and *B. lactis* in ice cream showed a decrease compared to the non‐encapsulated treatment Encapsulated *B. lactis* in ice cream showed a higher retention (1.2 ± 0.2 10^9^) than *L. casei* (2.5 ± 0.3 × 10^8^) at the end of 180 days of frozen storage	Homayouni et al. (2008)
Cheese	*Sacharomyces boulardii CDBB‐L‐1483 ATCC‐MYC‐797*	Inulin	Emulsification The aqueous phase containing 1% (w/v) sodium alginate, 0.05% (w/v) inulin, 0.05% (w/v) mucilage, and probiotics was added to the vegetable oil phase with constant agitation Gelation was conducted by adding glacial acetic acid	A greater viability of co‐encapsulated *S. boulardii* was shown in cheese compared to the unencapsulated form after storage of 30 days at 4°C The organoleptic properties of cheese were improved by the addition of the encapsulated probiotic with inulin, with more consumer acceptability	Zamora‐Vega et al. (2012)
Yoghurt	*Lactobacillus acidophilus LA*‐5	FOS	Extrusion and external gelation The solution of alginate + gelatin or alginate + gelatin + FOS with prebiotic was added into the CaCl_2_ solution at a flow rate of 0.19 and 0.12 mL/s, respectively 30 cm distance was maintained between the atomizer nozzle and the CaCl_2_ solution, and the microbeads were kept for 30 min for gelation	Microbeads formed using alginate + gelatin + FOS recorded the highest EE (99.28% ± 6.47%) and more than 80% of survival of probiotic in yoghurt after 28 days of storage at 4°C Co‐encapsulated *L. acidophilus* microbeads showed higher viability during storage in yoghurt and after exposure to digestive conditions	Silva Cristina et al. (2017)
Yoghurt	*Bifidobacterium longum* *LMG 13197*	Inulin	Freeze drying of mixed emulsions *B. longum* , inulin, poly‐vinyl‐alcohol, and dichloromethane, with Vegetal BM 297 AT O (food grade lipid) emulsion, were added to emulsified lactose in poly‐vinyl‐alcohol Homogenized mixed emulsions were kept for 5 h to evaporate dichloromethane. Freeze drying was carried out at −60°C for 72 h.	Encapsulated *B.longum* with vegetal‐inulin showed the greatest viability after exposure to simulated gastrointestinal fluids over 6 h and showed the highest survival in yoghurt during storage for 6 weeks at 4°C. The lowest pH reduction of fortified yoghurt was observed by vegetal inulin added microcapsules.	Amakiri and Thantsha (2016).
Yoghurt	*Lactococcus lactis* Gh1	Extract of *Synsepalum dulcificum* (miracle fruit)	Spray drying Feed solution: *L. lactis* pellets with 5% (w/v) of gum arabic (ratio 1:1) and *Synsepalum dulcificum* extract Inlet drying temperature: 130°C Outlet drying temperature: 60°C Flow rate: 35.25 mL/min	*S. dulcificum* seed extract added microcapsules showed the highest EE (99.27%) and the highest cell viability of 2.0 × 10^8^ CFU/mL at 2 h under the simulated gastric juice Yoghurt incorporated with microencapsulated *L. lactis* retained a higher viability (around 10^7^ CFU/mL) compared to non‐microencapsulated cells (around 10^5^ CFU/mL)	Fazilah et al. (2019)

As a summary, among the co‐encapsulation techniques reviewed, spray drying emerges as one of the most practical methods for large‐scale production due to its high productivity, cost‐effectiveness, and compatibility with a variety of wall materials. By incorporating protective agents, spray drying not only enhances probiotic survival during processing but also promotes symbiotic functionality. Freeze drying is another highly effective method, particularly for preserving probiotic viability, making it ideal for formulations requiring longer shelf life and precise structural integrity. Additionally, electro‐hydrodynamic processes offer superior encapsulation precision and the production of nano and microparticles, encasing the targeted delivery of probiotics and prebiotics.

Optimizing wall materials is crucial across these techniques to enhance encapsulation efficiency and ensure effective gastrointestinal release. A combined approach, such as using spray drying for mass production and electro‐hydrodynamics for specialized applications, represents an advanced and scalable strategy for delivering high‐quality co‐encapsulated prebiotics and probiotics with improved viability in the food and pharmaceutical industries.

## 8 Conclusion and Future Perspectives

Functional foods, emerging as an indirect method for delivering essential nutrients, are an ideal strategy for fortified foods containing co‐encapsulated probiotics and prebiotics, contributing to enhancing human gut health. Many studies have demonstrated a significant improvement in probiotic stability and viability when they are co‐encapsulated with prebiotics. In the food industry, significant attention has been given to fortifying dairy products such as yoghurt and cheese using co‐encapsulated probiotics and prebiotics. Even though the development of functional dairy foods has been extensively studied, particularly concerning probiotics and prebiotics, there is still a great deal of space for the development of new functional foods. This includes a wide range of food products such as dairy, meat, and fish products, bakery products, and vegetable and fruit‐based foods.

The success of co‐encapsulation of probiotics with prebiotics relies on several factors, including the chosen encapsulation technique, the resilience of probiotics to withstand processing stress, and the probiotic potential of core materials. The use of suitable wall materials not only improves encapsulation efficiency but also enhances probiotic viability during encapsulation, fortification processes, and storage. Thus, careful selection of co‐encapsulation techniques, operating conditions, and ensuring probiotic‐prebiotic compatibility are crucial for achieving a high co‐encapsulation yield and efficiency with high survivability. Several studies have investigated co‐encapsulation through freeze drying, extrusion, and spray‐drying techniques. Thermo‐tolerant probiotics are better suited for spray drying, while thermo‐unstable probiotics are more suitable in freeze drying, extrusion, and similar methods. The combination of polysaccharides and proteins is often employed in research to boost the wall's shielding ability, ensuring microcapsule stability and providing increased protection to core materials.

It is critical to develop and optimize co‐encapsulation technologies using innovative wall and core materials to enhance probiotic stability across diverse food matrices and processing conditions. Advanced techniques, such as incorporating nanomaterials and bioactive compounds, could improve protection and controlled release. Additionally, there is a need to create novel functional foods fortified with co‐encapsulated probiotics and prebiotics that offer enhanced health benefits while remaining economically viable. However, challenges in cost, scalability, and regulatory approval must be addressed to ensure the commercial success of co‐encapsulated functional foods.

## Author Contributions

**D. S. Shanuke:** data curation (equal), writing – original draft (equal). **Nilmini Buddhika D. P. Ranasinghage:** data curation (equal), writing – original draft (equal). **Ashinshana U. Illippangama:** conceptualization (equal), formal analysis (equal), supervision (equal), writing – review and editing (equal). **Jayani Kulathunga:** data curation (equal), supervision (equal), writing – review and editing (equal). **Mithila D. Bandara:** conceptualization (equal), data curation (equal), formal analysis (equal), funding acquisition (equal), investigation (equal), supervision (equal), writing – review and editing (equal).

## Conflicts of Interest

The authors declare no conflicts of interest.

## Data Availability

The authors have nothing to report.
